# Life Cycle Environmental Impacts of Wastewater-Derived Phosphorus Products: An Agricultural End-User Perspective

**DOI:** 10.1021/acs.est.2c00353

**Published:** 2022-07-07

**Authors:** Ka Leung Lam, Kimberly Solon, Mingsheng Jia, Eveline I. P. Volcke, Jan Peter van der Hoek

**Affiliations:** † Department of Water Management, Delft University of Technology, Stevinweg 1, Delft 2628 CN, The Netherlands; ‡ Division of Natural and Applied Sciences, Duke Kunshan University, 8 Duke Avenue, Kunshan, Jiangsu 215316, China; § BioCo Research Group, Department of Green Chemistry and Technology, Ghent University, Coupure Links 653, Gent 9000, Belgium; ∥ Waternet, Korte Ouderkerkerdijk 7, Amsterdam 1096 AC, The Netherlands

**Keywords:** life cycle assessment, environmental impacts, phosphorus recovery, wastewater, agricultural land application, end-user perspective, resource recovery

## Abstract

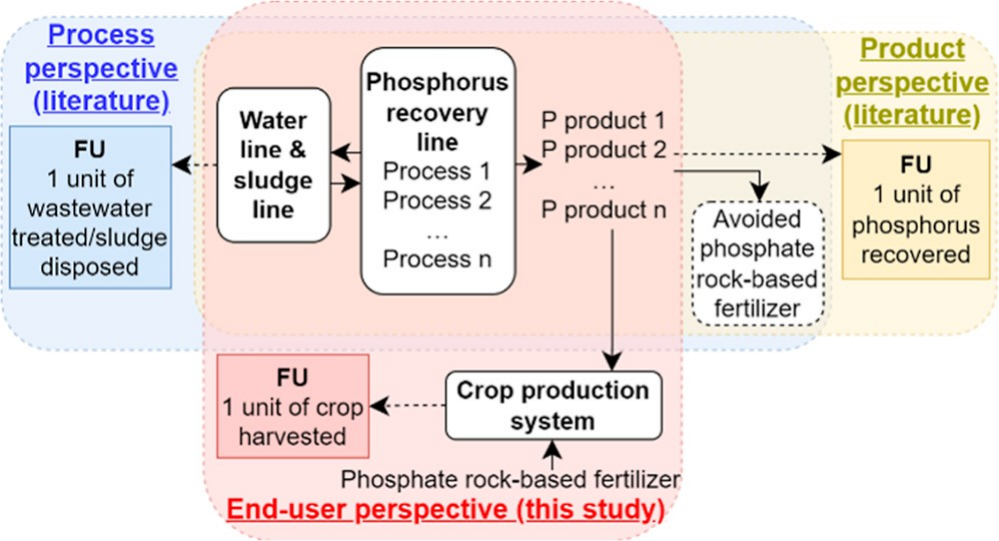

Recovering phosphorus from wastewater in more concentrated forms has potential to sustainably recirculate phosphorus from cities to agriculture. The environmental sustainability of wastewater-based phosphorus recovery processes or wastewater-derived phosphorus products can be evaluated using life cycle assessment (LCA). Many LCA studies used a *process perspective* to account for the impacts of integrating phosphorus recovery processes at wastewater treatment plants, while some used a *product perspective* to assess the impacts of producing wastewater-derived phosphorus products. We demonstrated the application of an *end-user perspective* by assessing life cycle environmental impacts of substituting half of the conventional phosphorus rock-based fertilizers used in three crop production systems with wastewater-derived phosphorus products from six recovery pathways (RPs). The consequential LCA results show that the substitution reduces global warming potential, eutrophication potential, ecotoxicity potential, and acidification potential of the assessed crop production systems in most RPs and scenarios. The *end-user perspective* introduced in this study can (i) complement with the *process perspective* and the *product perspective* to give a more holistic picture of environmental impacts along the “circular economy value chains” of wastewater-based resource recovery, (ii) enable systemwide assessment of wide uptake of wastewater-derived products, and (iii) draw attention to understanding the long-term environmental impacts of using wastewater-derived products.

## Abstract 2

This study shows life cycle environmental benefits of the agricultural use of wastewater-derived phosphorus products from the end-user perspective instead of the typical wastewater treatment plant perspective.

## Introduction

Resource recovery from wastewater is gaining increasing attention, especially phosphorus recovery.^1−3^ Phosphorus is essential for food production. Depleting phosphate rock reserves is becoming a driver for phosphorus recovery and reuse.^4^ In wastewater treatment plants (WWTPs), precipitation of struvite and Ca–P from sludge digester liquors are well-developed and economically feasible phosphorus recovery technologies, while wet chemical extraction from sewage sludge and sludge ashes can achieve a higher recovery rate of the influent phosphorus load.^5^ The recovered phosphorus products can potentially be used as fertilizers.^6^ Recovering phosphorus at WWTPs also has the benefits of reducing the potential of eutrophication in effluent-receiving waters and saving maintenance costs from uncontrolled phosphorus precipitation.^7−9^

Life cycle assessment (LCA) has been used to evaluate the potential environmental impacts of various wastewater-based phosphorus recovery and reuse opportunities.^10,11^ LCA can be used to compare technology alternatives, identify environmental hotspots, and understand environmental trade-offs.^12,13^ For instance, Amann et al.^14^ assessed the life cycle energy, global warming potential, and acidification potential of phosphorus recovery technologies from the liquid phase, sewage sludge, and sludge ashes. They found that recovery from the liquid phase (e.g., precipitation of struvite and Ca–P from sludge digester liquors) mostly has lower impacts on greenhouse gas (GHG) emissions and energy demand, though liquid phase recovery can only recover a fraction of the influent phosphorus load. In assessing both centralized and decentralized phosphorus recovery scenarios, Bradford-Hartke et al.^15^ showed that chemical-based phosphorus recovery generally has a net environmental burden as the benefits from avoided fertilizers cannot offset the burdens from increased resource inputs.

**Figure 1:**
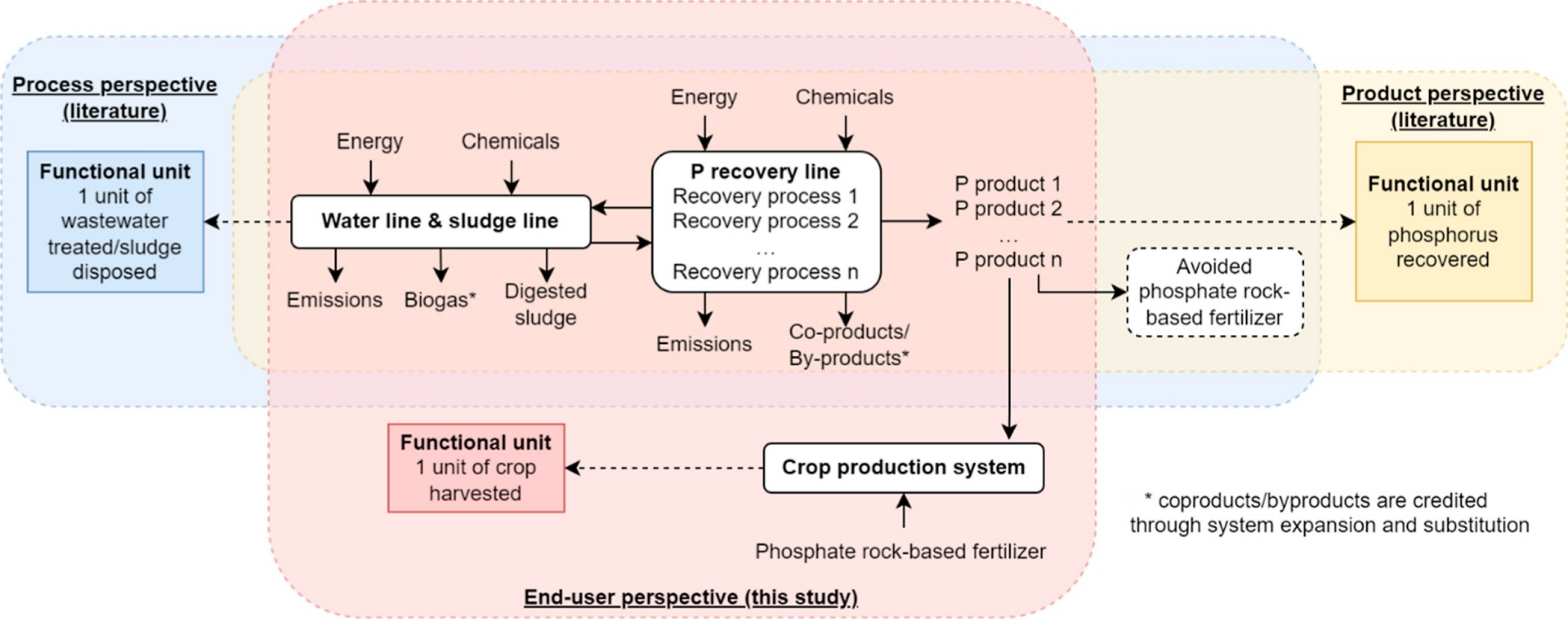
Process, product, and end-user perspectives of phosphorus recovery.

Most LCA studies related to wastewater-based phosphorus recovery focus their assessments on the primary functionality of wastewater treatment or sludge disposal, that is, applying a *process perspective* (Figure 1), in which phosphorus recovery is considered as an additional functionality (i.e., avoiding the use of a phosphate rock-base fertilizer). While the *process perspective* (also called the *waste management perspective*) of assessing environmental impacts is useful for the design and operation of a WWTP and its recovery process, the end users of the recovered phosphorus products do not know the life cycle environmental impacts of applying these recovered products to their product systems.^11^ Only few studies have quantified the environmental impacts of the wastewater-derived phosphorus products by applying a *product perspective* (Figure 1). Tonini et al.^16^ and Hörtenhuber et al.^17^ suggested that phosphorus products from incinerated sludge ashes have environmental advantages over conventional phosphate rock-based fertilizers. In this study, we extend the *product perspective* to an *end-user perspective* to investigate phosphorus recovery from the agricultural system’s point of view for the first time. To realize the total value of phosphorus recovery, end users must be considered early in the process of promoting phosphorus recovery.^7^ This can be contributed by *end-user perspective* LCAs. The resource recovery implications of these perspectives (with different functional units) will be discussed in detail.

This study assesses the life cycle environmental impacts of the agricultural use of wastewater-derived phosphorus products via six recovery pathways (RPs) in three crop production systems. Using phosphorus recovery as an example, this study demonstrates the application of the *end-user perspective* to develop a life cycle inventory and to understand the potential life cycle environmental consequences of substituting conventional inputs with recovered products at the end user’s product system. As more wastewater-derived products are becoming available, this perspective contributes toward understanding the potential systemic environmental consequences of a broader uptake of these products. The implications of conducting resource recovery LCA from an *end-user perspective*, compared to those from a *process* or *product perspective*, are discussed as well.

## Materials and Methods

### Goal and Scope

The primary goal of this study is to assess life cycle environmental impacts of substituting conventional phosphate rock-based fertilizers with wastewater-derived phosphorus products in crop production systems from the *end-user perspective* (Figure 1). Applying the *end-user perspective*, the system boundary encompasses a crop production system and a WWTP integrated with phosphorus recovery. When conducting LCA for their own crops, crop producers would like to know when they apply wastewater-derived phosphorus products, how they could get the inventory for these recovered products, and what could be the potential environmental consequences of using these recovered products instead of conventional phosphate rock-based fertilizers.

**Figure 2:**
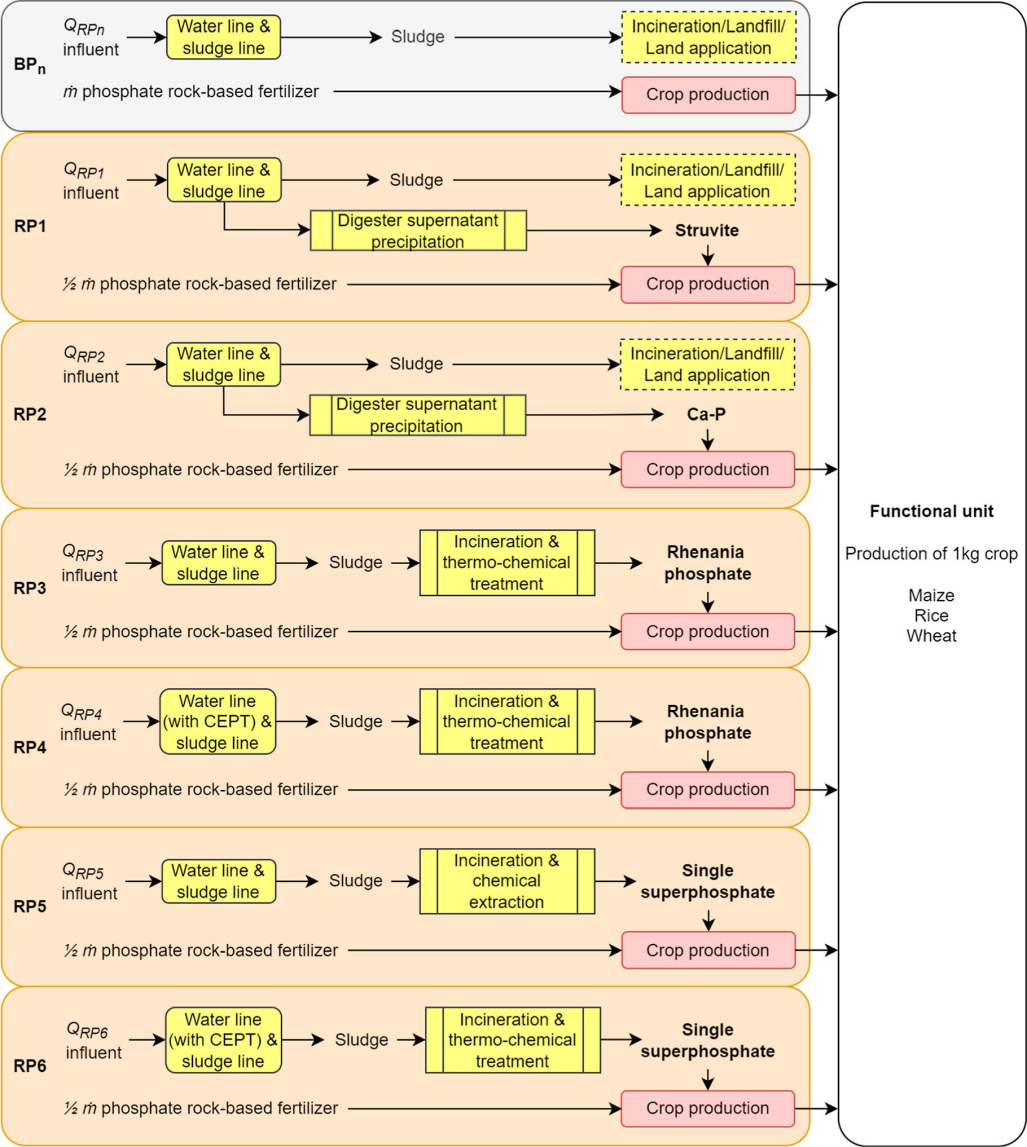
Baseline pathways (BP*n*) without P recovery and six possible P recovery pathways (RP1–RP6), differing in the recovered phosphorus products (i.e., struvite, Ca–P, rhenania phosphate-like product, or single superphosphate-like product) and the possible inclusion of CEPT in the treatment line. For all RPs, the wastewater-derived phosphorus product was assumed to substitute half (1/2 *ṁ*) of the conventional phosphate rock-based fertilizers used in the baseline pathways (BP*n*). Besides, all RPs share the same baseline for the crop production system, while each RP has its baseline for the wastewater treatment system. Three crop production systems (i.e., maize, rice, and wheat) were considered.

Phosphorus products can be recovered via different RPs at municipal WWTPs (Figure 2). In this study, the treatment plant is a typical plant with an activated sludge water line, a sludge line with anaerobic digestion for biogas production, and a phosphorus recovery line.^18^ Three major crop production systems are explored. For each crop, six RPs are compared based on a functional unit of producing 1 kg of that crop. The three crops are maize, rice, and wheat. Their cultivation using some of the wastewater-derived phosphorus products has been investigated in the literature.^19−23^ In addition, the life cycle inventory of these crop production systems is available in the Ecoinvent life cycle inventory database. This study is based loosely on the U.S. context—(i) it uses the U.S. national average maize, rice, and wheat production systems from the Ecoinvent database,^24^ (ii) it includes scenarios of typical sludge disposal methods in the U.S. (i.e., incineration, landfill, and land application),^25^ and (iii) it uses the Tool for Reduction and Assessment of Chemicals and Other Environmental Impacts (TRACI 2.1) developed by the U.S. Environmental Protection Agency as the impact assessment method.^26^

A “consequential” LCA approach^27^ is used to assess the potential environmental impacts of the decision of substituting half of the conventional phosphate rock-based fertilizers with wastewater-derived phosphorus products, that is, whether applying the wastewater-derived phosphorus products would generally or conditionally lead to net environmental benefits or not. Half substitution is assumed because previous studies have shown that blending slow-releasing wastewater-derived phosphorus products with conventional phosphate rock-based fertilizers would not restrict early season growth.^19^ Coproducts are being accounted for through system expansion (mainly biogas to avoid methane production).

### Life Cycle Inventory

#### Pathways and Scenarios

A life cycle inventory was built for six RPs (RP1–RP6) and their associated baseline pathways (BP*n*, where *n* is from 1 to 6, respectively) (Figure 2). In RP1, struvite is precipitated through the addition of magnesium hydroxide to the digester supernatant. In RP2, Ca–P, in the form of tricalcium phosphate, is precipitated through the addition of calcium hydroxide to the digester supernatant. In practice, struvite and Ca–P are the most common phosphorus forms recovered in the water line.^28,29^ In RP3, the sludge ashes from mono-incineration of digested sewage sludge undergoes a thermo-chemical treatment to yield a rhenania phosphate-like product. In RP5, the sludge ashes from mono-incineration of digested sewage sludge undergoes a chemical extraction process to yield single superphosphate and byproducts (i.e., calcium chloride and iron(III) chloride). Both RP4 and RP6 have the same recovery processes as RP3 and RP5, respectively, but RP4 and RP6 have chemically enhanced primary treatment (CEPT) in the water line to increase the phosphorus content in the sewage sludge. In practice, rhenania phosphate-like product and single superphosphate are common phosphorus forms recovered from the sludge ashes.^14,16^

Each RP has its associated baseline pathway. While all the RPs share the same baseline for the crop production system (i.e., the same phosphate rock-base fertilizer usage *ṁ*), each RP has its associated baseline for the wastewater treatment system (i.e., influent flowrate *Q*_RPn_). Because the yields of the recovered phosphorus product differ across various RPs, different flowrates of the influent are needed to yield the recovered phosphorus product in each RP. Each RP substitutes half of the amount of the phosphate rock-based fertilizer (1/2 *ṁ*) with its wastewater-derived phosphorus product, while the influent flowrate of each RP is the same as that of its associated baseline pathway. An illustration of Figure 2 with numbers for a given scenario can be found in the Supporting Information (Figures S1 and S2).

Each pathway was modeled considering three different influent wastewater compositions [i.e., concentrations of P, N, chemical oxygen demand (COD), etc.],^30^ three alternative sludge disposal methods (incineration, landfill, and land application), and three carbon intensity levels of grid electricity (low, medium, and high). For each crop, this results in 162 combinations of scenarios in total (i.e., 3^3^ = 27 scenarios/pathway × 6 pathways). The baseline pathways have no phosphorus recovery, but they have phosphorus recycling in scenarios of land applications of the digested sewage sludge (i.e., in other scenarios, the digested sludge is either incinerated or landfilled). The defined scenarios represent local factors of resource recovery facilities. These local factors can potentially influence the “embodied” environmental impacts of the wastewater-derived phosphorus products.

While this study has a baseline pathway for both the WWTP system and the crop production system, a baseline is not a must for the crop production system because one may be more interested in absolute impacts (i.e., of using recovered products) instead of relative impacts (i.e., of substituting conventional fertilizers with recovered products).

#### Inventory Development Overview

The key steps of this life cycle inventory phase are (i) to use a plant-wide modeling simulation to develop water line, sludge line, and recovery line (WWTP) foreground inventories for the six RPs and associated baseline pathways (detailed in the following paragraphs), (ii) to use literature data to develop recovery line (post-WWTP) foreground inventories for the four ash-based RPs (RP3–RP6), (iii) to use literature data and the Ecoinvent database to develop the inventories for crop production systems under all pathways, and (iv) to connect the upstream WWTP system and the downstream crop production system. A detailed workflow can be found in Section S1 of the Supporting Information.

The life cycle inventory is based on a plant-wide modeling simulation using a modified version of the Benchmark Simulation Model No. 2 (BSM2-PSFe),^18^ literature data,^16,31^ and the Ecoinvent database^24^ (Table 1).

**Table 1:** Overview of the Key Inventory

inventory category (Tables in the Supporting Information)	key parameters	sources
water and sludge lines (Tables S2–S4)	energy use, chemical use, emissions to water, biogas yield, sludge yield	modeling with BSM2-PSFe
recovery lines for RP1 and RP2 (Tables S2–S4)	energy use, chemical use, recovery yield	modeling with BSM2-PSFe
recovery lines for RP3, RP4, RP5, and RP6 (Tables S2–S4)	energy use, chemical use, material use, recovery yield	literature inventory
agronomic effectiveness (Table S5)	phosphorus content, bioavailability factor	literature inventory
sludge disposal by incineration, landfill, and land application (Table S10)	energy use, material use, emissions to soil, transportation	literature inventory
electricity supply with low, medium, or high GHG intensity (Table S11)	electricity use	Ecoinvent database
crop production for maize, rice, and wheat (Table S6)	substituted conventional fertilizers	Ecoinvent database
phosphate rock-based fertilizer supply (Table S11)	consumption rate	Ecoinvent database
chemical and material supply (Table S11)	consumption rate	Ecoinvent database

#### Water, Sludge, and Recovery Line Inventories

To model the dynamics of integrating phosphorus recovery into a WWTP, a plant-wide model was used. Plant-wide modeling and simulation is useful for evaluating the integration of resource recovery techniques in WWTPs.^32^ In the literature, only a few LCA studies used plant-wide models to build their foreground inventories,^33^ while most other LCA studies used static data from pilot-scale systems and multiple literature data sources.^34^ Plant-wide modeling enables the exploration of many different recovery scenarios, the consideration of the interaction of recovery processes with the rest of the WWTPs, the analysis of the effects of recovery on the overall plant performance, and the monitoring of the effluent discharge level over the simulated period.

A modified version of BSM2-PSFe^18,35^ was used to simulate the water line and the sludge line for the baseline pathways (BP*n*), the water line and the sludge line for all the RPs (RP1–RP6), and the recovery line (digester supernatant precipitation) for RP1 and RP2. BSM2 was designed for benchmarking the performance of WWTPs and testing control and operational strategies. BSM2-PSFe has the added capacity of modeling plant-wide phosphorus transformations. In this study, the results from steady-state simulations (1000 days) were used. The specific model outputs used for LCA include the electricity use; heating energy use; material use; biogas yield; struvite/Ca–P yield; sludge yield; and effluent P, N, and COD contents under various scenarios (i.e., influent pollutant concentration) and pathways (i.e., baseline, RP1 with struvite precipitation, RP2 with Ca–P precipitation, and RP4 and RP6 with CEPT). Since BSM2-PSFe does not model the downstream sludge disposal process, the recovery line for RP3–RP6 (sludge ash-based) was separately modeled with inventory from the literature.^15^

#### Other Inventories

The other key inventories include the maize grain production, rice (non-basmati) production, wheat grain production, sewage sludge disposal, bioavailability factor of recovered phosphorus products, electricity supply with three levels of the GHG emission intensity, phosphate rock-based fertilizer, and chemical and material use. They (together with any other background inventory) are taken from Ecoinvent 3.6, except for land application of the sludge^26^ and bioavailability of recovered phosphorus products.^15^

#### Impact on Nitrogen Pathways

The studied phosphorus RPs have direct and indirect impacts on nitrogen pathways. Struvite (RP1) contributes nitrogen nutrient directly for crop production, and it helps avoid a small fraction of conventional nitrogen fertilizers used (12–25% for the three crop production systems). On the other hand, since one-third of all the baseline scenarios include land application of the digested sludge (where nitrogen recycling occurs), reducing the amount of this sludge (RP1 and RP2) or directing all this sludge to incineration for ash-based phosphorus recovery (RP3–RP6) means more conventional nitrogen fertilizer is needed to compensate for less nitrogen recycling.

### Impact Assessment

TRACI 2.1 was used as the impact assessment method.^26^ In this study, we focused on assessing four impact categories—global warming potential, eutrophication potential, ecotoxicity potential, and acidification potential. They are impact categories that are most commonly assessed in wastewater-based nutrient recycling LCA studies.^11^ The LCA results are presented as the changes in impacts compared to those in the baseline scenarios.

### Sensitivity and Uncertainty

Sensitivity analysis is a common step in LCA for interpreting results and for identifying priorities for improved inventory data collection or impact assessment.^11^ It can be performed by testing key scenario assumptions one at a time.^16^ In this study, sensitivity analysis is partly embedded in the scenario modeling, where in each RP, we have a result set of 27 scenarios instead of a single deterministic result. In particular, it gives indications of how sensitive the results are to the three factors—influent pollutant concentration, sludge disposal method, and carbon intensity of grid electricity.

For uncertainty analysis, a Monte Carlo simulation was performed on the maize production system by propagating parameter uncertainties (Table S17). The uncertainty analysis result is presented as the probability of each resource RP having a lower impact potential than the baseline pathway. This resonates the objective of understanding whether applying the wastewater-derived phosphorus products would generally or conditionally lead to net environmental benefits or not. The uncertainty analysis is given in the Supporting Information.

## Results

### Life Cycle Environmental Impacts of Applying Wastewater-Derived Phosphorus Products

The LCA results are presented as the changes of life cycle environmental impacts compared to the baseline pathways (Figure 2). We assessed four common life cycle impact categories—global warming potential, eutrophication potential, ecotoxicity potential, and acidification potential.

Substituting half of the conventional phosphate rock-based fertilizers originally used in maize, rice, and wheat production systems with wastewater-derived phosphorus products from RP1, RP2, RP3, and RP4 reduces the assessed life cycle environmental impacts of these production systems in most scenarios [e.g., RP1 (wheat): 4–7% reduction in global warming potential, RP2 (wheat): 31–91% reduction in eutrophication potential]. The magnitude of change varies across the three crops. For instance, applying the rhenania phosphate-like product from RP4 could lead to 1–3 and 1–5% reduction in global warming potential for maize and wheat production, respectively. Essentially, the magnitude of change depends on the relative contribution of the fertilizer toward the overall life cycle environmental impact of a production system (i.e., having greater impacts on maize and wheat production systems).

RP3, RP5, and RP6 would increase global warming potential in all scenarios, while RP3 and RP6 are mostly favorable for reducing eutrophication potential and acidification potential. For RP5, the benefits of avoiding a conventional phosphorus rock-based fertilizer [plus calcium chloride and iron(III) chloride as byproducts] cannot outweigh the burdens of chemical inputs (mostly hydrochloric acid) and additional heating energy use for the recovery process. RP3 is particularly unfavorable for ecotoxicity potential because of the presence of heavy metals in the rhenania phosphate-like product, leading to emissions to soil, while RP4 generates a larger quantity of biogas (than RP3) which offsets the ecotoxicity impact of heavy metals. The use of a large quantity of chemicals in the recovery processes of RP5 and RP6 leads to their high ecotoxicity.

Eutrophication potential is the only impact category that is reduced in almost all pathways and scenarios. Plant-wide modeling shows that phosphorus recovery reduces the phosphorus content in the effluent and, therefore, its emissions to water. The recovery action diverts the phosphorus flow from the effluent phase to the recovered products.

### Influences of Local Factors on the Life Cycle Environmental Impacts

**Figure 3:**
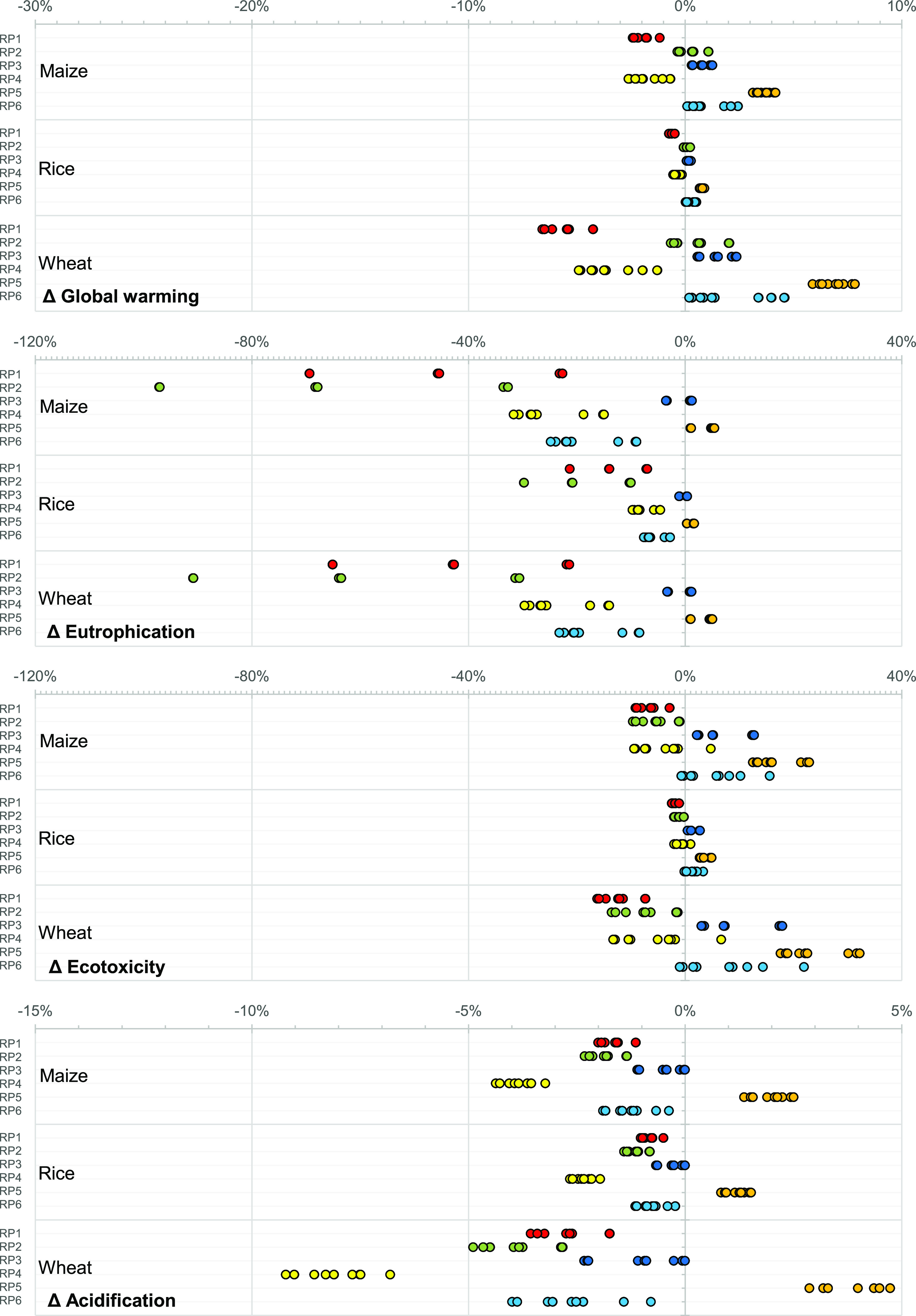
Changes in the global warming potential, eutrophication potential, ecotoxicity potential, and acidification potential in three different crop production systems after substituting half of the conventional phosphate rock-based fertilizers with wastewater-derived phosphorus products from six different RPs compared to the baseline pathway. Within a RP for a given crop, each dot is a scenario—one of the 27 combinations of the influent pollutant concentration, sludge disposal method, and carbon intensity of grid electricity. RP1: struvite from the digester supernatant; RP2: Ca–P from the digester supernatant; RP3 and RP4: rhenania phosphate-like product from the incinerated sludge ashes (RP4 with CEPT); RP5 and RP6: single superphosphate from the incinerated sludge ashes (RP6 with CEPT).

The three local factors assessed include (i) the level of pollutants in the influent at the WWTP, (ii) whether the digested sewage sludge is originally disposed by incineration, landfill, or land application, and (iii) the level of carbon intensity of local grid electricity. Within the RPs, some have higher variations of impacts across scenarios (Figure 3). Some of these higher-variation pathways include RP4 and RP6 in global warming potential, RP1 and RP2 in eutrophication potential, and RP4 and RP6 in ecotoxicity potential. They could be explained by exploring the life cycle inventory. In the cases of RP4 and RP6 in global warming potential, these two pathways have a greater net reduction in electricity use compared to other RPs. (They are therefore more sensitive to the level of the carbon intensity of local grid electricity.)

Within some RPs, the clustered dots indicate that one of those three local factors strongly influences the life cycle environmental impacts (e.g., the three clusters of dots for the global warming potential of RP3, the three clusters of dots for the eutrophication potential of RP1) (Figure 3). The level of pollutants in the influent at the WWTP is a strong determining factor for the eutrophication impacts. The original sewage sludge disposal approach has a strong influence on all the studied impact categories because of the very different sludge final disposal systems (Table S10) and the significant reduction in the amount of sludge destined for disposal in the recovery scenarios (particularly ash-based recovery in RP3–RP6) compared to the baseline (Figures S2 and S3). In contrast, the carbon intensity of grid electricity has a relatively insignificant influence. The breakdown of scenario results shown in Figure 3 is tabulated in the Supporting Information (Tables S12–S17).

The assessed scenarios represent only some of the local factors that could potentially influence the life cycle environment impacts “embodied” in the wastewater-derived phosphorus products. The results clearly imply that without an understanding of the “production system” of the wastewater-derived phosphorus products, end users are not capable of accounting for the life cycle environmental impacts of using these products.

### Comparison with Previous Studies

Previous studies have assessed the life cycle environmental impacts of integrating phosphorus recovery to WWTPs (*process perspective*)^33,36−38^ and of producing wastewater-derived phosphorus products (*product perspective*).^14−16,39,40^ The difference in system boundaries in these studies usually makes the results not directly comparable across studies.^11^ Nevertheless, *product perspective* studies are more comparable to the current study because both the *product perspective* and *end user perspective* emphasize on the recovered phosphorus products in their scope.

Some RPs assessed in this study were evaluated from a *product perspective* in the literature. In general, the results are consistent with those from the literature. For struvite recovery (like in RP1), Amann et al.^14^ showed that this liquid-phase recovery process leads to lower global warming potential (−0.5 to −1.4 kgCO_2_eq/PE/a) and acidification potential (−5.4 to −11.5 gSO_2_eq/PE/a) compared to those of a reference system without phosphorus recovery and with mono-incineration of the sewage sludge (i.e., in our study, −0.0052 to −0.0096 kgCO_2_eq/ 1 kg maize and −0.044 to −0.071 gSO_2_eq/ 1 kg maize). In addition, Amann et al.^14^ showed that Ca–P recovery (like in RP2) has higher global warming potential and acidification potential than the reference system. Linderholm et al.^39^ suggested that an ash-based recovered phosphorus product (like in RP3) has a much higher global warming potential than the precipitated struvite (like in RP1). Tonini et al.^16^ assessed a range of phosphorus recovery approaches from waste feedstock, including the recovery from municipal sewage sludge ashes as a rhenania phosphate-like product (like in RP3 and RP4) and single superphosphate (like in RP5 and RP6). Their study suggested that both products have a lower global warming potential, and a rhenania phosphate-like product has higher ecotoxicity compared to that of rock phosphate, while other impact categories are similar between these products and rock phosphate.

## Discussion

### Resource Recovery Implications of LCA with Different Perspectives

LCAs of wastewater-based resource recovery could generally be categorized as the *process perspective*, the *product perspective*, and the *end-user perspective* (Figure 1). The *process perspective* (also called the *waste management perspective*) evaluates the integration of resource recovery processes into conventional WWTPs. This perspective often aims to quantify and optimize the influence of resource recovery processes on the overall environmental performance of WWTPs. The focus is on the primary functionality of wastewater treatment and sludge disposal. The *product perspective* shifts attention from WWTPs to wastewater-derived products. This perspective often aims to evaluate and compare potential environmental impacts of the wastewater-derived products derived from different recovery approaches. The *end-user perspective* extends on the *product perspective*. It centers on how the application of wastewater-derived products impacts the overall environmental performance of the end users’ product system (i.e., in this study, the end user is the agricultural sector using wastewater-derived phosphorus products). One major difference between the *product perspective* and the *end-user perspective* is that the *product perspective* typically credits the recovered phosphorus products for avoiding an assumed conventional phosphate-rocked fertilizer production, while the *end-user perspective* only implicitly considers this when there is a baseline of using a conventional fertilizer (i.e., it is not considered for a new crop production system).

This study demonstrates the application of the *end-user perspective* for developing a life cycle inventory and to conduct LCAs of “downstream” product systems that utilize wastewater-derived products as inputs. The *end-user perspective* can serve three major purposes.

#### Complementing with Process and Product Perspectives

The *end-user perspective* can complement with the *process perspective* and the *product perspective* to give a more holistic picture of environmental impacts along the “circular economy value chains” of wastewater-based resource recovery. For WWTPs or water resource recovery facilities (WRRFs), phosphorus recovery (like any other resource recovery) can be a trade-off between recovery efficiency, environmental impacts, and economic revenues from the final products.^41^ The *process*/*product perspective* LCAs help WRRFs understand part of this trade-off.

On the other hand, the end users of wastewater-derived products are concerned with how the application of wastewater-derived products would influence the overall economic and environmental impacts of their product systems. The *end-user perspective* LCAs of wastewater-based resource recovery can be performed to address the environmental concern. To realize the total value of phosphorus recovery (and other forms of resource recovery as well), end users must be considered early in the process.^7^ By engaging end users in the early stage, WRRFs will also benefit from having access to better downstream inventories to improve the quality of their *process/product perspective* LCAs. Ultimately, conducting a LCA with these three perspectives (i.e., three types of functional units) on the same system encompassing both the WRRF (i.e., producer) and the end user (i.e., consumer) can enable a more holistic understanding of the environmental consequences of resource recovery. It is because the *end-user perspective* could be used to answer different questions such as what could be the impacts of using these recovered phosphorus products instead of conventional phosphate rock-based fertilizers? How much would this recovered phosphorus product contribute to the environmental benefit or burden of my crop production system?

#### Understanding Systemwide Impacts of Using Wastewater-Derived Products

The *end-user perspective* is essential to evaluate systemwide environmental impacts of wide uptake of wastewater-derived products at downstream product systems. This type of evaluation cannot be achieved from the *process*/*product perspective* because WRRFs would mostly have limited information on which downstream inputs (e.g., conventional fertilizers) would eventually be substituted by the wastewater-derived products (e.g., wastewater-derived phosphorus products). In addition, end users would likely to have better knowledge on the expected or actual performance and the limitations of these wastewater-derived phosphorus products.

LCA has been widely used for assessing environmental impacts of producing different agricultural commodities. The use of fertilizers and nutrient losses (P and N) are major contributors to environmental impacts.^42^ There are limited wastewater-derived product life cycle inventories that are transparent enough and directly useable by the agricultural sector. Using the *end-user perspective* could ensure that wastewater-derived product life cycle inventories are built with the end user in mind.

This study shows that all the studied RPs would reduce the amount of the sludge that was originally destined for landfill, incineration, or land application. As we modeled the baseline of sludge disposal in this study, the shift in the fate of the sludge has consequentially contributed to the results significantly. The fate of waste is not typically considered in the *product perspective* LCA. In order to better understand the systemwide impacts of circular economy, a better consideration of the fate of waste is needed.

#### Drawing More Attention to Long-Term Impacts and Effectiveness

The *end-user perspective* has an advantage of drawing more attention to the need of understanding the long-term environmental impacts and agronomic effectiveness of applying wastewater-derived phosphorus products (e.g., soil context,^43^ phosphorus uptake,^21^ and contaminants^44^). This long-term emission and effectiveness of these products is an important inventory, as identified by some field studies.^21,45^ As more wastewater-derived products are made available, it becomes more important to understand the environmental impacts of the “use phase” and the “disposal phase” (for some type of products) of these products, instead of only the “production phase” that the *process/product perspective* focused on.

### Future Outlook

Despite the value of *end-user perspective* LCA, it remains challenging for end users to derive inventories for the wastewater-derived products they use. This study shows that the LCA results can be sensitive to local factors of resource recovery facilities. For example, when the sewage sludge is diverted to mono-incineration for ash-based phosphorus recovery, the original destination of sludge disposal (i.e., landfill, incineration, or land application) can influence the results substantially. Other local factors such as the facility scale^10,46^ and recovered product transport distance^47^ are also potentially influential. Without a thorough understanding of the “upstream” resource recovery facility and the baseline system, the end users cannot robustly quantify the environmental impacts of their decision on using wastewater-derived products. This is especially critical when the original inputs to be substituted contribute a significant portion of life cycle environmental impacts of the end user’s product system. Therefore, WRRFs need to build transparent inventories in a way that is also useable by the end user.

Future LCA studies of the *end-user perspective* on phosphorus recovery can consider four areas of advancement. First, they can consider scenarios for which multiple wastewater-derived phosphorus products and other recovered products are produced. Second, they can address the data gap in the long-term crop-specific field performance (e.g., agronomic effectiveness and phosphorus leaching) of wastewater-derived phosphorus products to improve the overall quality of the life cycle inventory.^21,45^ Third, national average maize, rice, and wheat production systems in the U.S. were used directly from the Ecoinvent database without any modifications. LCA of an actual crop production system using wastewater-derived phosphorus products would be very valuable. Fourth, while this study used a consequential approach^27^ in developing the life cycle inventory, the *end-user perspective* does not constraint the choice of the LCA approach. Attributional approaches with different allocation methods could be tested for developing the inventory (e.g., without “zero burden assumption” for waste feedstock^47,48^ or partitioning^49^).

## Supporting Information Available

Detailed method; life cycle inventory; impact assessment results; uncertainty analysis; and sensitivity analysis (PDF)
