# Supplementary material for: Life Cycle Environmental Impacts of Wastewater-Derived Phosphorus Products: An Agricultural End-User Perspective
Source: Environ Sci Technol. 2022 Jul 7;56(14):10289–98. doi: 10.1021/acs.est.2c00353 (PMC9301919; doi:10.1021/acs.est.2c00353)
Supplement: Supplementary file 1 — es2c00353_si_001.pdf [file es2c00353_si_001.pdf]

Supporting Information

Life cycle environmental impacts of wastewater-derived phosphorus products: an agricultural end-user perspective

*Ka Leung Lam *, †, ‡, Kimberly Solon §, Mingsheng Jia §, Eveline I. P. Volcke §, Jan Peter van der Hoek †, ∥*

† Department of Water Management, Delft University of Technology, Stevinweg 1, 2628 CN, Delft, The Netherlands.
‡ Division of Natural and Applied Sciences, Duke Kunshan University, 8 Duke Avenue, Kunshan, Jiangsu, 215316, China.
§ BioCo Research Group, Department of Green Chemistry and Technology, Ghent University, Coupure Links 653, 9000 Gent, Belgium.
∥ Waternet, Korte Ouderkerkerdijk 7, 1096 AC, Amsterdam, The Netherlands.

* Corresponding Author. Email: k.l.lam@tudelft.nl, kaleung.lam@dukekunshan.edu.cn

Number of pages: 21
Number of tables: 19
Number of figures: 3

Table of Contents

S1. Workflow 2
S2. Inventories 3
S3. Results 12
S4. Monte Carlo simulation 18
S5. Sensitivity analysis 20
References 21

S1. Workflow

The major workflow is as follows:

1. The three influent pollutant levels were defined as inputs for BSM2-PSFe (Table S1). This represents one of three scenario dimensions (the other two defined in Step #7).
2. BSM2-PSFe simulation for its default typical wastewater treatment plant was performed in a steady state mode until the 1000th day simulation, where the results were used. The default run is for the baseline, RP3 and RP5. Struvite recovery modules and Ca-P recovery modules were activated for RP1 and RP2 respectively, while chemically enhanced primary treatment (CEPT) module was activated for RP4 and RP6.
3. BSM2-PSFe simulation outputs formed the inventory for **Water line**, **Sludge line**, **Recovery line (WWTP)** in Tables S2-S4 (each table for one of the three influent pollutant levels). For **Recovery line (post-WWTP)**, inputs for mono-incineration and ash-based recovery (RP3-RP6), and outputs from ash-based recovery (RP3-RP6) were derived by scaling up inventory from Tonini *et al.* (2019) based on the "Average sludge production (dry weight)" from the Sludge line.
4. To link the water resource recovery facility system to the crop production system, we need to know the phosphorus content and agronomic effectiveness of recovered phosphorus products (Table S5). These factors were mostly sourced from literature.
5. For the crop production system, existing process inventories for maize, rice and wheat production in the US were used directly from Ecoinvent 3.6. The only modification applied to these crop production inventories is that the input of conventional phosphate fertilizer reduces by half for RP1 to RP6 (for RP1, the input of nitrogen fertilizer also reduces accordingly based on the nitrogen content of struvite applied) (Table S6). In each RP, the quantity of recovered product (i.e., struvite, Ca-P, rhenania phosphate-like product, single superphosphate-like product) needed to substitute the halved conventional phosphate fertilizer depends on its phosphorus content and agronomic effectiveness (Table S5).
6. Once the foreground inventory for the water resource recovery facility system (Step #3) and crop production system (Step #6) were ready, we started building the LCA model in SimaPro 9.1. Each recovered product was defined as the difference between RP and BP in Tables S2-S4 (i.e., RP1 recovered product inventory = RP1 inventory – BP inventory). The output quantity of each recovered product (via one of the six RPs) is highlighted in grey in Tables S2-S4. These numbers were inputted directly to the fields of "*Outputs to technosphere: Products and co-products*" when defining these recovered products in SimaPro.
7. The two other scenario dimensions (sludge disposal methods and carbon intensity levels of grid electricity) were defined in SimaPro platform. Sludge disposal method scenarios require linking the disposed sludge output to the corresponding disposal processes (Table S10), while the carbon intensity levels of grid electricity scenarios require linking the electricity input to the corresponding electricity supply processes (Table S11). With that, we have in total 27 scenarios for each recovered product via one of the six RPs (Table S7).
8. After defining all six recovered phosphorus products in SimaPro, we could define the three crop production systems (Table S6). For each crop, we defined 162 recovery scenarios (i.e., 27 scenarios/pathway × 6 pathways) plus a baseline. It is done simply by using Table S6 inventory and the already-defined recovered phosphorus products in SimaPro.
9. Other inventories (Table S8, Table S10) were added to the corresponding processes in SimaPro.

S2. Inventories

Influent composition

The wastewater has a typical composition of raw municipal wastewater with minor contributions from industries. Three influent pollutant concentration levels (i.e., "low", "low-medium" and "medium") were defined. The flowrates are similar and only the concentrations of the components are varying. The "low-medium" level corresponds to the BSM2-PSFe[1] default influent composition. The "low" and "medium" levels were derived from the "low-medium" level using a factor of 0.6 and 1.17, respectively, for each influent state variable (except for fermentation products, S_A) to ensure agreement with typical concentration values and ratios found in Volcke *et al.* (2020),[2] after which the parameter values in Table S1 were calculated from the influent state variables. The default influent flow rate of 20,935m^3/d of BSM2-PSFe was used.

Table S1 Values of wastewater parameters according to "low", "low-medium" and "medium" levels.

**Parameter**	**Unit**	**Low**	**Low-Medium** BSM2-PSFe default[1]	**Medium**
Flowrate	$m^3.d^{-1}$	20935.15		
COD	$g.m^{-3}$	424.8	669.6	773.7
BOD_5	$g.m^{-3}$	227.0	353.5	407.2
Nitrogen	$g.m^{-3}$	31.0	51.6	60.4
Phosphorus	$g.m^{-3}$	6.0	10.0	15.0
VSS	$g.m^{-3}$	201.5	335.3	392.2
TSS	$g.m^{-3}$	238.2	374.7	432.7

Table S2 Inventory of water line, sludge line and recovery line for baseline pathway (BP) and recovery pathways 1 to 6 (RP1-RP6) for a reference influent average flow rate of 20,935m^3/d for 'low' level of influent pollutants

	Unit	Pathways						
		BP	RP1	RP2	RP3	RP4	RP5	RP6
**Inputs**								
**1. Water line** [a]								
Average aeration energy	kWh/d	2651.9	2510.9	2552.3	2651.9	1956.4	2651.9	1956.4
Average pumping energy	kWh/d	452.7	452.7	453.4	452.7	433.7	452.7	433.7
Average mixing energy	kWh/d	1008	1008	1008	1008	1008	1008	1008
Average iron addition	kg Fe/d	-	-	-	-	250	-	250
**2. Sludge line** [a]								
Average heating energy	kWh/d	4460.1	4369.2	4371.0	4460.1	3872.1	4460.1	3872.1
**3a. Recovery line (WWTP)** [a]								
Average magnesium addition	kg/d	-	36.45	-	-	-	-	-
Average sodium addition	kg/d	-	0.23	6.90	-	-	-	-
Average calcium addition	kg/d	-	-	84.17	-	-	-	-
**3b. Recovery line (Post-WWTP)** [b]								
Mono-incineration								
Natural gas	kWh/d	-	-	-	111.31	134.73	-	-
Silica	kg/d	-	-	-	146.17	176.93	-	-
Ash-based recovery I								
Ca(OH)2	kg/d	-	-	-	20.08	24.31	-	-
Electricity	kWh/d	-	-	-	100.41	121.54	-	-
Heat	kWh/d	-	-	-	433.88	525.18	-	-
NaOH	kg/d	-	-	-	16.74	20.26	-	-
NaSO4	kg/d	-	-	-	412.81	499.67	-	-
Water	kg/d	-	-	-	323.55	391.64	-	-
Ash-based recovery II								
Electricity	kWh/d	-	-	-	-	-	33.47	40.51
HCl	kg/d	-	-	-	-	-	371.53	449.71
Heat	kWh/d	-	-	-	-	-	2270.4	2748.2
**Outputs**								
**1. Water line** [a]								
Emissions to water: average total P load	kg P/d	29.82	14.73	15.08	29.82	12.46	29.82	12.46
Emissions to water: average total N load	kg N/d	120.25	123.75	126.93	120.25	108.82	120.25	108.82
Emissions to water: average total COD load	kg COD/d	569.22	587.51	586.45	569.22	593.38	569.22	593.38
**2. Sludge line** [a]								
Average sludge production (dry weight)	kg/d	2625.2	2445.9	2447.3	2625.2	3177.6	2625.2	3177.6
Biogas production	kWh/d	8970.3	9195.2	9186.7	8970.3	10467.1	8970.3	10467.1
**3a. Recovery line (WWTP)** [a]								
Average P recovery	kg P/d	-	46.10	43.22	-	-	-	-
**3b. Recovery line (Post-WWTP)** [b]								
Ash-based recovery I								
Average P recovery	kg P/d	-	-	-	93.97	110.99	-	-
Average recovery residue to final disposal	kg/d	-	-	-	1.56	1.56	-	-
Ash-based recovery II								
Average P recovery	kg P/d	-	-	-	-	-	94.91	112.10
Average recovery residue to final disposal	kg/d	-	-	-	-	-	6.02	7.29
Average calcium chloride recovery	kg/d	-	-	-	-	-	74.75	90.48
Average iron (III) chloride recovery	kg/d	-	-	-	-	-	45.74	55.37

[a] Outputs from BSM2-PSFe simulation
[b] Estimates from scaling ash-based recovery inventory from Tonini *et al.* (2019)[3] with average sludge production (dry weight)
All sludge produced in RP3-RP6 is mono-incinerated (highlighted in red).

Table S3 Inventory of water line, sludge line and recovery line for baseline pathway (BP) and recovery pathways 1 to 6 (RP1-RP6) for a reference influent average flow rate of 20,935m^3/d for 'low-medium' level of influent pollutants

	Unit	Pathways						
		BP	RP1	RP2	RP3	RP4	RP5	RP6
**Inputs**								
**1. Water line [a]**								
Average aeration energy	kWh/d	4178.8	3964.6	4051.3	4178.8	3507.1	4178.8	3507.1
Average pumping energy	kWh/d	452.7	455.2	453.5	452.7	452.7	452.7	452.7
Average mixing energy	kWh/d	1008	1008	1008	1008	1008	1008	1008
Average iron addition	kg Fe/d	-	-	-	-	250	-	250
**2. Sludge line [a]**								
Average heating energy	kWh/d	4887.9	4817.2	4821.1	4887.9	4308.3	4887.9	4308.3
**3a. Recovery line (WWTP) [a]**								
Average magnesium addition	kg/d	-	60.75	-	-	-	-	-
Average sodium addition	kg/d	-	0.23	7.59	-	-	-	-
Average calcium addition	kg/d	-	-	140.28	-	-	-	-
**3b. Recovery line (Post-WWTP) [b]**								
Mono-incineration								
Natural gas	kWh/d	-	-	-	165.27	194.59	-	-
Silica	kg/d	-	-	-	217.03	255.53	-	-
Ash-based recovery I								
Ca(OH)2	kg/d	-	-	-	29.82	35.11	-	-
Electricity	kWh/d	-	-	-	149.09	175.54	-	-
Heat	kWh/d	-	-	-	644.23	758.51	-	-
NaOH	kg/d	-	-	-	24.85	29.26	-	-
NaSO4	kg/d	-	-	-	612.94	721.67	-	-
Water	kg/d	-	-	-	480.41	565.63	-	-
Ash-based recovery II								
Electricity	kWh/d	-	-	-	-	-	49.70	58.51
HCl	kg/d	-	-	-	-	-	551.64	649.50
Heat	kWh/d	-	-	-	-	-	3371.2	3969.2
**Outputs**								
**1. Water line [a]**								
Emissions to water: average total P load	kg P/d	75.06	20.93	21.91	75.06	18.12	75.06	18.12
Emissions to water: average total N load	kg N/d	190.84	181.68	189.42	190.84	220.15	190.84	220.15
Emissions to water: average total COD load	kg COD/d	846.33	855.87	854.30	846.33	776.54	846.33	776.54
**2. Sludge line [a]**								
Average sludge production (dry weight)	kg/d	3897.9	3738.6	3743.6	3897.9	4589.3	3897.9	4589.3
Biogas production	kWh/d	14287	14489	14459	14287	18325	14287	18325
**3a. Recovery line (WWTP) [a]**								
Average P recovery	kg P/d	-	77.15	71.31	-	-	-	-
**3b. Recovery line (Post-WWTP) [b]**								
Ash-based recovery I								
Average P recovery	kg P/d	-	-	-	131.76	187.58	-	-
Average recovery residue to final disposal	kg/d	-	-	-	2.32	2.73	-	-
Ash-based recovery II								
Average P recovery	kg P/d	-	-	-	-	-	133.08	189.46
Average recovery residue to final disposal	kg/d	-	-	-	-	-	8.95	10.53
Average calcium chloride recovery	kg/d	-	-	-	-	-	110.99	130.68
Average iron (III) chloride recovery	kg/d	-	-	-	-	-	67.92	79.97

[a] Outputs from BSM2-PSFe simulation
[b] Estimates from scaling ash-based recovery inventory from Tonini *et al.* (2019)[3] with average sludge production (dry weight)
All sludge produced in RP3-RP6 is mono-incinerated (highlighted in red).

Table S4 Inventory of water line, sludge line and recovery line for baseline pathway (BP) and recovery pathways 1 to 6 (RP1-RP6) for a reference influent average flow rate of 20,935m^3/d for 'medium' level of influent pollutants

	Unit	Pathways						
		BP	RP1	RP2	RP3	RP4	RP5	RP6
**Inputs**								
**1. Water line** [a]								
Average aeration energy	kWh/d	4830.0	4664.9	4776.9	4830.0	4132.9	4830.0	4132.9
Average pumping energy	kWh/d	452.7	455.9	453.5	452.7	452.7	452.7	452.7
Average mixing energy	kWh/d	1008	1008	1008	1008	1008	1008	1008
Average iron addition	kg Fe/d	-	-	-	-	250	-	250
**2. Sludge line** [a]								
Average heating energy	kWh/d	5068.6	5078.3	5062.4	5068.6	4450.9	5068.6	4450.9
**3a. Recovery line (WWTP)** [a]								
Average magnesium addition	kg/d	-	91.13	-	-	-	-	-
Average sodium addition	kg/d	-	0.23	8.05	-	-	-	-
Average calcium addition	kg/d	-	-	210.10	-	-	-	-
**3b. Recovery line (Post-WWTP)** [b]								
Mono-incineration								
Natural gas	kWh/d	-	-	-	188.65	220.97	-	-
Silica	kg/d	-	-	-	247.72	290.17	-	-
Ash-based recovery I								
Ca(OH)2	kg/d	-	-	-	34.04	39.87	-	-
Electricity	kWh/d	-	-	-	170.18	199.34	-	-
Heat	kWh/d	-	-	-	735.34	861.33	-	-
NaOH	kg/d	-	-	-	28.36	33.22	-	-
NaSO4	kg/d	-	-	-	699.62	819.49	-	-
Water	kg/d	-	-	-	548.35	642.30	-	-
Ash-based recovery II								
Electricity	kWh/d	-	-	-	-	-	56.73	66.45
HCl	kg/d	-	-	-	-	-	629.66	737.54
Heat	kWh/d	-	-	-	-	-	3847.9	4507.2
**Outputs**								
**1. Water line** [a]								
Emissions to water: average total P load	kg P/d	162.67	34.98	48.34	162.67	93.66	162.67	93.66
Emissions to water: average total N load	kg N/d	220.99	202.65	216.05	220.99	262.64	220.99	262.64
Emissions to water: average total COD load	kg COD/d	962.63	959.75	960.64	962.63	876.63	962.63	876.63
**2. Sludge line** [a]								
Average sludge production (dry weight)	kg/d	4449.1	4432.2	4399.7	4449.1	5211.4	4449.1	5211.4
Biogas production	kWh/d	16481	16397	16431	16481	21090	16481	21090
**3a. Recovery line (WWTP)** [a]								
Average P recovery	kg P/d	-	115.93	105.35	-	-	-	-
**3b. Recovery line (Post-WWTP)** [b]								
Ash-based recovery I								
Average P recovery	kg P/d	-	-	-	148.06	215.71	-	-
Average recovery residue to final disposal	kg/d	-	-	-	2.65	3.10	-	-
Ash-based recovery II								
Average P recovery	kg P/d	-	-	-	-	-	149.54	217.87
Average recovery residue to final disposal	kg/d	-	-	-	-	-	10.21	11.96
Average calcium chloride recovery	kg/d	-	-	-	-	-	126.69	148.39
Average iron (III) chloride recovery	kg/d	-	-	-	-	-	77.53	90.81

[a] Outputs from BSM2-PSFe simulation
[b] Estimates from scaling ash-based recovery inventory from Tonini *et al.* (2019)[3] with average sludge production (dry weight)
All sludge produced in RP3-RP6 is mono-incinerated (highlighted in red).

Table S5 Phosphorus content and agronomic effectiveness of recovered phosphorus products

Recovered phosphorus products	Phosphorus content	Bioavailability	Remark
Struvite (RP1)	0.126	1	Assumed
Ca-P (RP2)	0.200	0.7	Based on Terman *el al.* (1958)[4] and Römer & Steingrobe (2018)[5]
Rhenania phosphate-like product (RP3-RP4)	0.074	0.9	Tonini *et al.* (2019)[3]
Single superphosphate-like product (RP5-RP6)	0.087	1	Tonini *et al.* (2019)[3]

Table S6 Inventory of crop production systems in baseline pathway (BP) and recovery pathways (RPs)

	Unit	Pathways						
		BP	RP1	RP2	RP3	RP4	RP5	RP6
**Maize**								
Maize production process [a]	kg	1	1	1	1	1	1	1
Phosphate fertilizer [b]	kg	0.005848	0.002924	0.002924	0.002924	0.002924	0.002924	0.002924
Ammonium nitrate [c]	kg	0.0048927	0.0043155	0.0048927	0.0048927	0.0048927	0.0048927	0.0048927
Struvite	kg	-	0.010111	-	-	-	-	-
Ca-P	kg	-	-	0.009128	-	-	-	-
Rhenania phosphate-like product	kg	-	-	-	0.019159	0.019159	-	-
Single superphosphate-like product	kg	-	-	-	-	-	0.014666	0.014666
**Rice**								
Rice production process [d]	kg	1	1	1	1	1	1	1
Phosphate fertilizer [b]	kg	0.0043758	0.0021879	0.0021879	0.0021879	0.0021879	0.0021879	0.0021879
Nitrogen fertilizer [e]	kg	0.0017123	0.0012804	0.0017123	0.0017123	0.0017123	0.0017123	0.0017123
Struvite	kg	-	0.007566	-	-	-	-	-
Ca-P	kg	-	-	0.00683	-	-	-	-
Rhenania phosphate-like product	kg	-	-	-	0.014336	0.014336	-	-
Single superphosphate-like product	kg	-	-	-	-	-	0.010974	0.010974
**Wheat**								
Wheat production process [f]	kg	1	1	1	1	1	1	1
Phosphate fertilizer [b]	kg	0.018503	0.009252	0.009252	0.009252	0.009252	0.009252	0.009252
Nitrogen fertilizer [e]	kg	0.0072404	0.0054141	0.0072404	0.0072404	0.0072404	0.0072404	0.0072404
Struvite	kg	-	0.031991	-	-	-	-	-
Ca-P	kg	-	-	0.028882	-	-	-	-
Rhenania phosphate-like product	kg	-	-	-	0.060618	0.060618	-	-
Single superphosphate-like product	kg	-	-	-	-	-	0.04640	0.04640

[a] Using "Maize grain {US}| production | Conseq, U", less "Phosphate fertiliser, as P2O5 {GLO}| market for | Conseq, U" and "Ammonium nitrate, as N {GLO}| market for | Conseq, U" [Ecoinvent 3.6]

[b] Using "Phosphate fertiliser, as P2O5 {GLO}| market for | Conseq, U" [Ecoinvent 3.6]

[c] Using "Ammonium nitrate, as N {GLO}| market for | Conseq, U" [Ecoinvent 3.6]

[d] Using "Rice, non-basmati {US}| rice production, non-basmati | Conseq, U", less "Phosphate fertiliser, as P2O5 {GLO}| market for | Conseq, U" and "Nitrogen fertiliser, as N {GLO}| market for | Conseq, U" [Ecoinvent 3.6]

[e] Using "Nitrogen fertiliser, as N {GLO}| market for | Conseq, U" [Ecoinvent 3.6]

[f] Using "Wheat grain {US}| wheat production | Conseq, U", less "Phosphate fertiliser, as P2O5 {GLO}| market for | Conseq, U" and "Nitrogen fertiliser, as N {GLO}| market for | Conseq, U" [Ecoinvent 3.6]

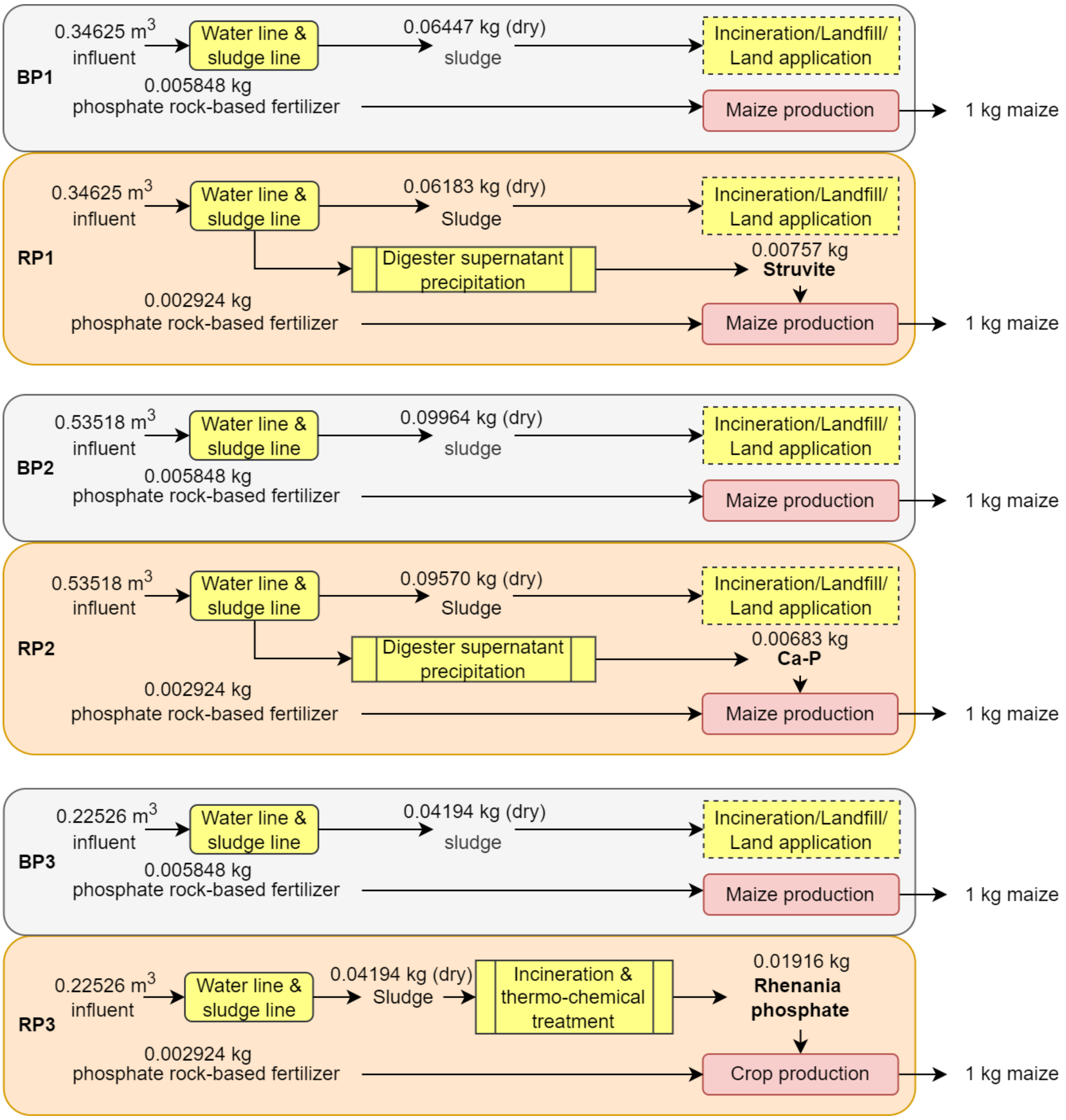

Figure S1 One set of inventories for 1 kg maize production (RP1 to RP3, "low-medium" pollutant level scenario)

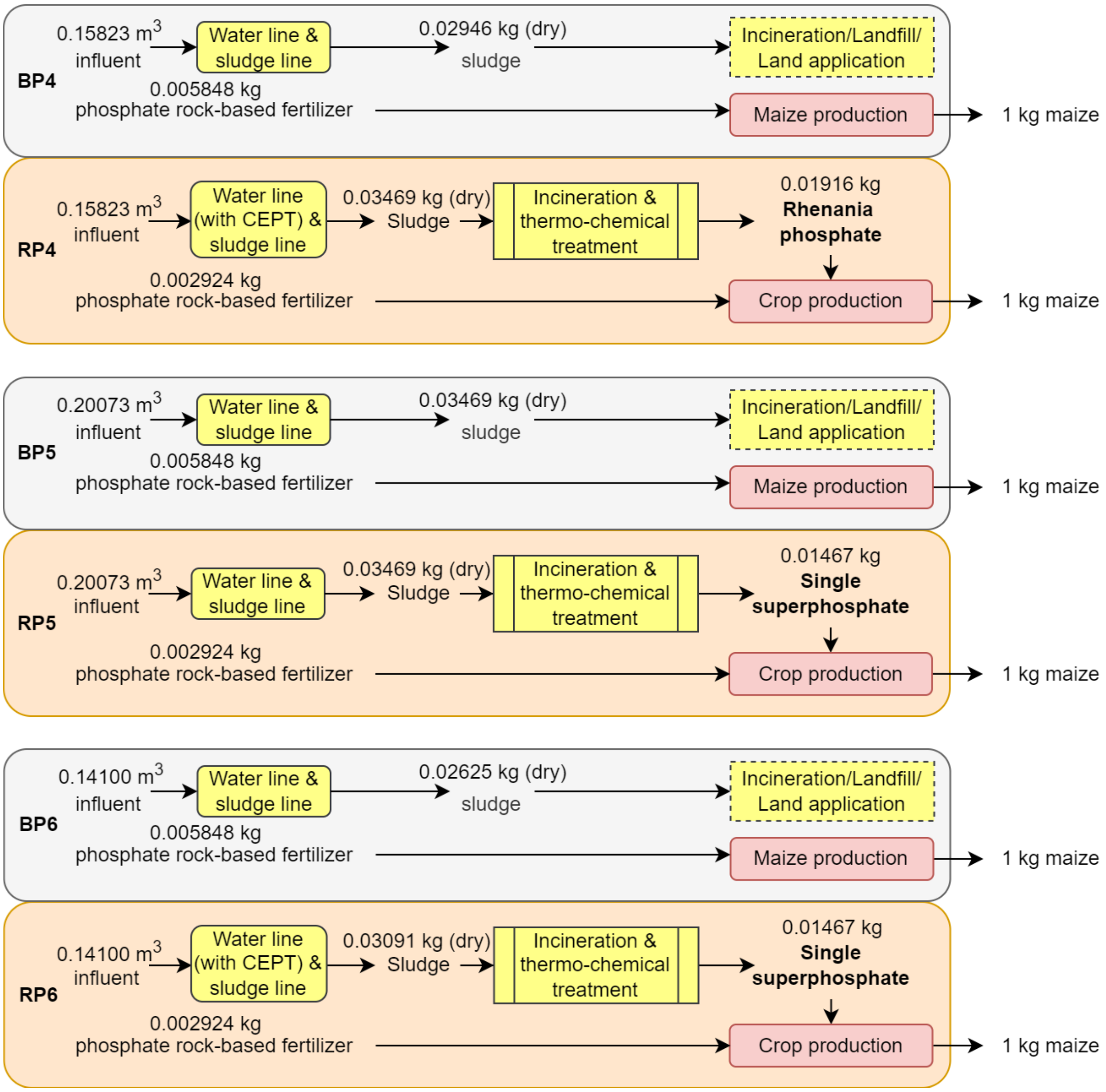

Figure S2 One set of inventories for 1 kg maize production (RP4 to RP6, "low-medium" pollutant level scenario)

Table S7 Scenario combinations

Recovered product	Influent pollutant concentration	Sludge disposal	Carbon intensity of electricity
RPn-S01	Low	Incineration	Low
RPn-S02	Low	Incineration	Medium
RPn-S03	Low	Incineration	High
RPn-S04	Low	Landfill	Low
RPn-S05	Low	Landfill	Medium
RPn-S06	Low	Landfill	High
RPn-S07	Low	Land application	Low
RPn-S08	Low	Land application	Medium
RPn-S09	Low	Land application	High
RPn-S10	Low-medium	Incineration	Low
RPn-S11	Low-medium	Incineration	Medium
RPn-S12	Low-medium	Incineration	High
RPn-S13	Low-medium	Landfill	Low
RPn-S14	Low-medium	Landfill	Medium
RPn-S15	Low-medium	Landfill	High
RPn-S16	Low-medium	Land application	Low
RPn-S17	Low-medium	Land application	Medium
RPn-S18	Low-medium	Land application	High
RPn-S19	Medium	Incineration	Low
RPn-S20	Medium	Incineration	Medium
RPn-S21	Medium	Incineration	High
RPn-S22	Medium	Landfill	Low
RPn-S23	Medium	Landfill	Medium
RPn-S24	Medium	Landfill	High
RPn-S25	Medium	Land application	Low
RPn-S26	Medium	Land application	Medium
RPn-S27	Medium	Land application	High

Table S8 Emissions to soil from recovered phosphorus products

Heavy metal	Unit	RP1	RP2	RP3 [a]	RP4 [a]	RP5 [a]	RP6 [a]
As	mg kg^{-1} TS	<1	<1	4	4	<1	<1
Cd	mg kg^{-1} TS	<1	<1	0	0	<1	<1
Cr	mg kg^{-1} TS	<1	<1	127	127	5	5
Cu	mg kg^{-1} TS	<1	<1	601	601	5	5
Hg	mg kg^{-1} TS	<1	<1	1	1	<1	<1
Ni	mg kg^{-1} TS	<1	<1	56	56	<1	<1
Pb	mg kg^{-1} TS	<1	<1	60	60	<1	<1
Zn	mg kg^{-1} TS	<1	<1	1737	1737	3	4

[a] Tonini *et al.* (2019)[3]

Table S9 Transportation

Item	Unit	Distance
Recovered products to land application	km	400
Sludge to incineration	km	100
Sludge to land application	km	100
Sludge to landfill	km	100

Table S10 Sludge disposal

Items	Unit	Value
Dewatered sludge water content	-	72%
Land application (per 1 ton of dry sludge)[6]		
Outputs to technosphere: Avoided products: Nitrogen fertiliser, as N {RoW}| monoammonium phosphate production | APOS, U	kg	1.93
Outputs to technosphere: Avoided products: Nitrogen fertiliser, as N {RoW}| diammonium phosphate production | APOS, U	kg	2.76
Outputs to technosphere: Avoided products: Phosphate fertiliser, as P2O5 {RER}| single superphosphate production | Conseq, U	kg	13.94
Inputs from nature: Carbon dioxide, in air	kg	192
Emissions to Soil: Arsenic	kg	0.01
Emissions to Soil: Copper	kg	0.314
Emissions to Soil: Chromium	kg	0.047
Emissions to Soil: Cadmium	kg	0.002
Emissions to Soil: Mercury	kg	0.001
Emissions to Soil: Molybdenum	kg	0.006
Emissions to Soil: Nickel	kg	0.015
Emissions to Soil: Lead	kg	0.069
Emissions to Soil: Selenium	kg	0.001
Emissions to Soil: Zinc	kg	0.696
Incineration[7]		
Digester sludge {GLO}| treatment of digester sludge, municipal incineration | Conseq, U		
Landfill[7]		
Process-specific burdens, sanitary landfill {RoW}| market for process-specific burdens, sanitary landfill | Conseq, U		

Table S11 Background inventory from Ecoinvent 3.6[7]

Item	Remark
Electricity, medium voltage {MRO, US only}| market for | Conseq, U	Representing high carbon intensity electricity
Electricity, medium voltage {WECC, US only}| market for | Conseq, U	Representing medium carbon intensity electricity
Electricity, medium voltage {NPCC, US only}| market for | APOS, U	Representing low carbon intensity electricity
Methane, 96% by volume, from biogas, low pressure, at user {RoW}| production | Conseq, U	Avoided products
Magnesium oxide {GLO}| market for | Conseq, U	Representing magnesium hydroxide production
Transport, freight, lorry, unspecified {GLO}| market group for transport, freight, lorry, unspecified | Conseq, U	
Sodium hydroxide, without water, in 50% solution state {GLO}| market for | Conseq, U	
Lime, hydrated, loose weight {RoW}| market for lime, hydrated, loose weight | Conseq, U	
Sodium sulfate, anhydrite {RER}| sodium sulfate production, from natural sources | Conseq, U	
Tap water {Europe without Switzerland}| market for | Conseq, U	
Silica sand {GLO}| market for | Conseq, U	
Hydrochloric acid, without water, in 30% solution state {RER}| market for | Conseq, U	
Calcium chloride {RER}| market for calcium chloride | Conseq, U	Avoided products
Iron(III) chloride, without water, in 14% iron solution state {GLO}| market for | Conseq, U	Avoided products

S3. Results

Table S12 Impact assessment results for Figure 3 – RP1-RP3 for maize

ID	Influent pollutant concentration	Sludge disposal	Carbon intensity of electricity	RP1				RP2				RP3			
				GWP	EP	ET	AP	GWP	EP	ET	AP	GWP	EP	ET	AP
S01	Low	Incineration	Low	-2.18%	-23.19%	-9.19%	-1.85%	-0.19%	-33.54%	-9.65%	-2.14%	1.15%	-3.36%	2.49%	-0.12%
S02	Low	Incineration	Medium	-2.17%	-23.19%	-9.19%	-1.85%	-0.19%	-33.54%	-9.65%	-2.13%	1.14%	-3.36%	2.49%	-0.12%
S03	Low	Incineration	High	-2.18%	-23.19%	-9.19%	-1.85%	-0.20%	-33.54%	-9.65%	-2.14%	1.15%	-3.36%	2.49%	-0.12%
S04	Low	Landfill	Low	-2.42%	-22.68%	-8.06%	-2.01%	-0.35%	-32.74%	-7.78%	-2.32%	0.30%	0.86%	12.36%	-1.11%
S05	Low	Landfill	Medium	-2.41%	-22.68%	-8.06%	-2.00%	-0.35%	-32.74%	-7.78%	-2.32%	0.30%	0.86%	12.36%	-1.11%
S06	Low	Landfill	High	-2.42%	-22.68%	-8.06%	-2.01%	-0.36%	-32.74%	-7.78%	-2.32%	0.30%	0.86%	12.36%	-1.11%
S07	Low	Land application	Low	-2.36%	-22.65%	-8.96%	-1.93%	-0.27%	-32.69%	-9.14%	-2.21%	0.72%	1.12%	5.20%	-0.52%
S08	Low	Land application	Medium	-2.36%	-22.65%	-8.96%	-1.93%	-0.27%	-32.69%	-9.14%	-2.21%	0.72%	1.12%	5.20%	-0.52%
S09	Low	Land application	High	-2.37%	-22.65%	-8.96%	-1.93%	-0.28%	-32.69%	-9.14%	-2.21%	0.72%	1.12%	5.20%	-0.52%
S10	Low-medium	Incineration	Low	-1.76%	-45.69%	-6.45%	-1.55%	0.39%	-68.31%	-5.50%	-1.78%	1.24%	-3.50%	2.22%	-0.03%
S11	Low-medium	Incineration	Medium	-1.76%	-45.69%	-6.45%	-1.55%	0.39%	-68.31%	-5.50%	-1.78%	1.23%	-3.50%	2.22%	-0.03%
S12	Low-medium	Incineration	High	-1.76%	-45.69%	-6.45%	-1.55%	0.39%	-68.31%	-5.50%	-1.78%	1.24%	-3.50%	2.22%	-0.03%
S13	Low-medium	Landfill	Low	-1.82%	-45.41%	-5.79%	-1.62%	0.30%	-67.89%	-4.52%	-1.88%	0.34%	0.97%	12.66%	-1.07%
S14	Low-medium	Landfill	Medium	-1.81%	-45.41%	-5.79%	-1.62%	0.31%	-67.89%	-4.52%	-1.88%	0.34%	0.97%	12.66%	-1.07%
S15	Low-medium	Landfill	High	-1.82%	-45.41%	-5.79%	-1.62%	0.30%	-67.89%	-4.52%	-1.88%	0.34%	0.97%	12.66%	-1.07%
S16	Low-medium	Land application	Low	-1.79%	-45.39%	-6.27%	-1.58%	0.35%	-67.87%	-5.23%	-1.82%	0.79%	1.24%	5.08%	-0.45%
S17	Low-medium	Land application	Medium	-1.79%	-45.39%	-6.27%	-1.58%	0.35%	-67.87%	-5.23%	-1.82%	0.79%	1.24%	5.08%	-0.45%
S18	Low-medium	Land application	High	-1.79%	-45.39%	-6.27%	-1.58%	0.35%	-67.87%	-5.23%	-1.82%	0.79%	1.24%	5.08%	-0.45%
S19	Medium	Incineration	Low	-1.17%	-69.39%	-2.90%	-1.14%	1.09%	-97.13%	-1.19%	-1.34%	1.26%	-3.54%	2.14%	0.00%
S20	Medium	Incineration	Medium	-1.17%	-69.39%	-2.90%	-1.14%	1.09%	-97.13%	-1.19%	-1.34%	1.26%	-3.54%	2.14%	0.00%
S21	Medium	Incineration	High	-1.17%	-69.39%	-2.90%	-1.14%	1.09%	-97.13%	-1.19%	-1.34%	1.26%	-3.54%	2.14%	0.00%
S22	Medium	Landfill	Low	-1.17%	-69.37%	-2.85%	-1.14%	1.07%	-97.04%	-0.97%	-1.36%	0.36%	1.00%	12.75%	-1.06%
S23	Medium	Landfill	Medium	-1.17%	-69.37%	-2.85%	-1.14%	1.07%	-97.04%	-0.97%	-1.36%	0.35%	1.00%	12.75%	-1.06%
S24	Medium	Landfill	High	-1.18%	-69.37%	-2.85%	-1.14%	1.07%	-97.04%	-0.97%	-1.36%	0.36%	1.00%	12.75%	-1.06%
S25	Medium	Land application	Low	-1.17%	-69.36%	-2.88%	-1.14%	1.08%	-97.03%	-1.13%	-1.35%	0.81%	1.27%	5.05%	-0.43%
S26	Medium	Land application	Medium	-1.17%	-69.36%	-2.88%	-1.14%	1.08%	-97.03%	-1.13%	-1.35%	0.81%	1.27%	5.05%	-0.43%
S27	Medium	Land application	High	-1.17%	-69.36%	-2.88%	-1.14%	1.08%	-97.03%	-1.13%	-1.35%	0.81%	1.27%	5.05%	-0.42%

GWP: Global warming potential, EP: Eutrophication potential, ET: Ecotoxicity, AP: Acidification potential

Table S13 Impact assessment results for Figure 3 – RP4-RP6 for maize

ID	Influent pollutant concentration	Sludge disposal	Carbon intensity of electricity	RP4				RP5				RP6			
				GWP	EP	ET	AP	GWP	EP	ET	AP	GWP	EP	ET	AP
S01	Low	Incineration	Low	-0.69%	-18.76%	-3.60%	-3.23%	3.89%	0.99%	12.53%	2.24%	2.43%	-12.36%	8.16%	-0.37%
S02	Low	Incineration	Medium	-0.68%	-18.76%	-3.61%	-3.23%	3.89%	0.99%	12.53%	2.24%	2.44%	-12.36%	8.16%	-0.37%
S03	Low	Incineration	High	-0.69%	-18.76%	-3.60%	-3.23%	3.89%	0.99%	12.53%	2.24%	2.43%	-12.36%	8.16%	-0.38%
S04	Low	Landfill	Low	-1.40%	-15.18%	4.75%	-4.06%	3.13%	4.78%	21.41%	1.36%	1.80%	-9.18%	15.61%	-1.12%
S05	Low	Landfill	Medium	-1.39%	-15.18%	4.75%	-4.06%	3.13%	4.78%	21.41%	1.36%	1.81%	-9.18%	15.61%	-1.11%
S06	Low	Landfill	High	-1.40%	-15.18%	4.75%	-4.06%	3.13%	4.78%	21.41%	1.36%	1.80%	-9.18%	15.61%	-1.12%
S07	Low	Land application	Low	-1.05%	-14.97%	-1.31%	-3.56%	3.51%	5.01%	14.96%	1.89%	2.12%	-8.99%	10.21%	-0.67%
S08	Low	Land application	Medium	-1.04%	-14.97%	-1.31%	-3.56%	3.51%	5.01%	14.96%	1.89%	2.13%	-8.99%	10.21%	-0.67%
S09	Low	Land application	High	-1.05%	-14.97%	-1.31%	-3.56%	3.51%	5.01%	14.96%	1.89%	2.11%	-8.99%	10.21%	-0.67%
S10	Low-medium	Incineration	Low	-1.95%	-30.70%	-9.19%	-3.64%	4.10%	1.08%	13.21%	2.44%	0.73%	-23.90%	-0.30%	-1.24%
S11	Low-medium	Incineration	Medium	-1.94%	-30.70%	-9.19%	-3.64%	4.10%	1.08%	13.21%	2.44%	0.73%	-23.90%	-0.30%	-1.24%
S12	Low-medium	Incineration	High	-1.95%	-30.70%	-9.19%	-3.64%	4.10%	1.08%	13.21%	2.44%	0.72%	-23.90%	-0.30%	-1.24%
S13	Low-medium	Landfill	Low	-2.57%	-27.56%	-1.85%	-4.37%	3.30%	5.06%	22.52%	1.51%	0.17%	-21.10%	6.24%	-1.89%
S14	Low-medium	Landfill	Medium	-2.57%	-27.56%	-1.85%	-4.37%	3.30%	5.06%	22.52%	1.51%	0.17%	-21.10%	6.24%	-1.89%
S15	Low-medium	Landfill	High	-2.58%	-27.56%	-1.85%	-4.37%	3.30%	5.06%	22.52%	1.51%	0.17%	-21.10%	6.24%	-1.89%
S16	Low-medium	Land application	Low	-2.26%	-27.37%	-7.18%	-3.93%	3.70%	5.30%	15.77%	2.07%	0.45%	-20.93%	1.50%	-1.50%
S17	Low-medium	Land application	Medium	-2.26%	-27.37%	-7.18%	-3.93%	3.70%	5.30%	15.77%	2.07%	0.45%	-20.93%	1.50%	-1.50%
S18	Low-medium	Land application	High	-2.26%	-27.37%	-7.18%	-3.93%	3.70%	5.30%	15.77%	2.07%	0.44%	-20.93%	1.50%	-1.50%
S19	Medium	Incineration	Low	-1.99%	-31.69%	-9.43%	-3.55%	4.17%	1.11%	13.45%	2.50%	0.65%	-24.83%	-0.71%	-1.19%
S20	Medium	Incineration	Medium	-1.99%	-31.69%	-9.43%	-3.55%	4.17%	1.11%	13.45%	2.50%	0.66%	-24.83%	-0.71%	-1.19%
S21	Medium	Incineration	High	-1.99%	-31.69%	-9.43%	-3.55%	4.17%	1.11%	13.45%	2.50%	0.65%	-24.83%	-0.71%	-1.19%
S22	Medium	Landfill	Low	-2.61%	-28.57%	-2.14%	-4.28%	3.36%	5.15%	22.90%	1.56%	0.10%	-22.05%	5.78%	-1.84%
S23	Medium	Landfill	Medium	-2.61%	-28.57%	-2.14%	-4.28%	3.36%	5.15%	22.90%	1.56%	0.10%	-22.05%	5.78%	-1.84%
S24	Medium	Landfill	High	-2.61%	-28.57%	-2.14%	-4.28%	3.36%	5.15%	22.90%	1.56%	0.10%	-22.05%	5.78%	-1.84%
S25	Medium	Land application	Low	-2.30%	-28.39%	-7.43%	-3.84%	3.77%	5.40%	16.04%	2.13%	0.38%	-21.88%	1.07%	-1.45%
S26	Medium	Land application	Medium	-2.30%	-28.39%	-7.43%	-3.84%	3.76%	5.40%	16.04%	2.13%	0.38%	-21.88%	1.07%	-1.45%
S27	Medium	Land application	High	-2.30%	-28.39%	-7.43%	-3.84%	3.77%	5.40%	16.04%	2.13%	0.38%	-21.88%	1.07%	-1.45%

GWP: Global warming potential, EP: Eutrophication potential, ET: Ecotoxicity, AP: Acidification potential

Table S14 Impact assessment results for Figure 3 – RP1-RP3 for rice

				RP1				RP2				RP3			
**ID**	**Influent pollutant concentration**	**Sludge disposal**	**Carbon intensity of electricity**	GWP	EP	ET	AP	GWP	EP	ET	AP	GWP	EP	ET	AP
S01	Low	Incineration	Low	-0.70%	-7.17%	-2.47%	-0.94%	-0.04%	-10.28%	-2.07%	-1.30%	0.24%	-1.03%	0.53%	-0.08%
S02	Low	Incineration	Medium	-0.69%	-7.17%	-2.47%	-0.94%	-0.04%	-10.28%	-2.07%	-1.30%	0.24%	-1.03%	0.53%	-0.08%
S03	Low	Incineration	High	-0.70%	-7.17%	-2.47%	-0.94%	-0.04%	-10.28%	-2.07%	-1.30%	0.24%	-1.03%	0.53%	-0.08%
S04	Low	Landfill	Low	-0.75%	-7.01%	-2.23%	-1.03%	-0.08%	-10.04%	-1.67%	-1.41%	0.06%	0.26%	2.65%	-0.67%
S05	Low	Landfill	Medium	-0.74%	-7.01%	-2.23%	-1.03%	-0.07%	-10.04%	-1.67%	-1.41%	0.06%	0.26%	2.65%	-0.67%
S06	Low	Landfill	High	-0.75%	-7.01%	-2.23%	-1.03%	-0.08%	-10.04%	-1.67%	-1.41%	0.06%	0.26%	2.65%	-0.67%
S07	Low	Land application	Low	-0.73%	-7.00%	-2.42%	-0.98%	-0.06%	-10.02%	-1.96%	-1.34%	0.15%	0.34%	1.11%	-0.31%
S08	Low	Land application	Medium	-0.73%	-7.00%	-2.42%	-0.98%	-0.06%	-10.02%	-1.96%	-1.34%	0.15%	0.34%	1.11%	-0.31%
S09	Low	Land application	High	-0.74%	-7.00%	-2.42%	-0.98%	-0.06%	-10.02%	-1.96%	-1.34%	0.15%	0.34%	1.11%	-0.31%
S10	Low-medium	Incineration	Low	-0.61%	-14.06%	-1.88%	-0.75%	0.08%	-20.94%	-1.18%	-1.08%	0.26%	-1.07%	0.47%	-0.02%
S11	Low-medium	Incineration	Medium	-0.61%	-14.06%	-1.88%	-0.75%	0.08%	-20.94%	-1.18%	-1.08%	0.26%	-1.07%	0.47%	-0.02%
S12	Low-medium	Incineration	High	-0.61%	-14.06%	-1.88%	-0.75%	0.08%	-20.94%	-1.18%	-1.08%	0.26%	-1.07%	0.47%	-0.02%
S13	Low-medium	Landfill	Low	-0.62%	-13.98%	-1.74%	-0.79%	0.06%	-20.81%	-0.97%	-1.14%	0.07%	0.30%	2.71%	-0.65%
S14	Low-medium	Landfill	Medium	-0.62%	-13.98%	-1.74%	-0.79%	0.07%	-20.81%	-0.97%	-1.14%	0.07%	0.30%	2.71%	-0.65%
S15	Low-medium	Landfill	High	-0.62%	-13.98%	-1.74%	-0.79%	0.06%	-20.81%	-0.97%	-1.14%	0.07%	0.30%	2.71%	-0.65%
S16	Low-medium	Land application	Low	-0.61%	-13.97%	-1.85%	-0.77%	0.07%	-20.80%	-1.12%	-1.11%	0.17%	0.38%	1.09%	-0.27%
S17	Low-medium	Land application	Medium	-0.61%	-13.97%	-1.85%	-0.77%	0.07%	-20.80%	-1.12%	-1.10%	0.17%	0.38%	1.09%	-0.27%
S18	Low-medium	Land application	High	-0.61%	-13.97%	-1.85%	-0.77%	0.07%	-20.80%	-1.12%	-1.11%	0.17%	0.38%	1.09%	-0.27%
S19	Medium	Incineration	Low	-0.48%	-21.33%	-1.12%	-0.50%	0.23%	-29.77%	-0.25%	-0.81%	0.27%	-1.09%	0.46%	0.00%
S20	Medium	Incineration	Medium	-0.48%	-21.33%	-1.12%	-0.50%	0.23%	-29.77%	-0.25%	-0.81%	0.27%	-1.09%	0.46%	0.00%
S21	Medium	Incineration	High	-0.48%	-21.33%	-1.12%	-0.50%	0.23%	-29.77%	-0.25%	-0.81%	0.27%	-1.09%	0.46%	0.00%
S22	Medium	Landfill	Low	-0.48%	-21.32%	-1.11%	-0.50%	0.23%	-29.74%	-0.21%	-0.83%	0.08%	0.31%	2.73%	-0.64%
S23	Medium	Landfill	Medium	-0.48%	-21.32%	-1.11%	-0.50%	0.23%	-29.74%	-0.21%	-0.83%	0.08%	0.31%	2.73%	-0.64%
S24	Medium	Landfill	High	-0.48%	-21.32%	-1.11%	-0.50%	0.23%	-29.74%	-0.21%	-0.83%	0.08%	0.31%	2.73%	-0.64%
S25	Medium	Land application	Low	-0.48%	-21.32%	-1.12%	-0.50%	0.23%	-29.74%	-0.24%	-0.82%	0.17%	0.39%	1.08%	-0.26%
S26	Medium	Land application	Medium	-0.48%	-21.32%	-1.12%	-0.50%	0.23%	-29.74%	-0.24%	-0.82%	0.17%	0.39%	1.08%	-0.26%
S27	Medium	Land application	High	-0.48%	-21.32%	-1.12%	-0.50%	0.23%	-29.74%	-0.24%	-0.82%	0.17%	0.39%	1.08%	-0.26%

GWP: Global warming potential, EP: Eutrophication potential, ET: Ecotoxicity, AP: Acidification potential

Table S15 Impact assessment results for Figure 3 – RP4-RP6 for rice

				RP4				RP5				RP6			
**ID**	**Influent pollutant concentration**	**Sludge disposal**	**Carbon intensity of electricity**	GWP	EP	ET	AP	GWP	EP	ET	AP	GWP	EP	ET	AP
S01	Low	Incineration	Low	-0.15%	-5.75%	-0.77%	-1.96%	0.83%	0.30%	2.68%	1.36%	0.52%	-3.79%	1.75%	-0.23%
S02	Low	Incineration	Medium	-0.14%	-5.75%	-0.77%	-1.96%	0.83%	0.30%	2.68%	1.36%	0.52%	-3.79%	1.75%	-0.23%
S03	Low	Incineration	High	-0.15%	-5.75%	-0.77%	-1.96%	0.83%	0.30%	2.68%	1.36%	0.52%	-3.79%	1.75%	-0.23%
S04	Low	Landfill	Low	-0.30%	-4.65%	1.02%	-2.47%	0.67%	1.47%	4.58%	0.83%	0.38%	-2.81%	3.34%	-0.68%
S05	Low	Landfill	Medium	-0.30%	-4.65%	1.02%	-2.47%	0.67%	1.47%	4.58%	0.83%	0.38%	-2.81%	3.34%	-0.68%
S06	Low	Landfill	High	-0.30%	-4.65%	1.02%	-2.47%	0.67%	1.47%	4.58%	0.83%	0.38%	-2.81%	3.34%	-0.68%
S07	Low	Land application	Low	-0.22%	-4.59%	-0.28%	-2.16%	0.75%	1.54%	3.20%	1.15%	0.45%	-2.76%	2.19%	-0.41%
S08	Low	Land application	Medium	-0.22%	-4.59%	-0.28%	-2.16%	0.75%	1.54%	3.20%	1.15%	0.45%	-2.76%	2.19%	-0.41%
S09	Low	Land application	High	-0.22%	-4.59%	-0.28%	-2.16%	0.75%	1.54%	3.20%	1.15%	0.45%	-2.76%	2.19%	-0.41%
S10	Low-medium	Incineration	Low	-0.41%	-9.41%	-1.97%	-2.21%	0.87%	0.33%	2.83%	1.48%	0.15%	-7.32%	-0.06%	-0.76%
S11	Low-medium	Incineration	Medium	-0.41%	-9.41%	-1.97%	-2.21%	0.87%	0.33%	2.83%	1.48%	0.16%	-7.32%	-0.06%	-0.76%
S12	Low-medium	Incineration	High	-0.41%	-9.41%	-1.97%	-2.21%	0.87%	0.33%	2.83%	1.48%	0.15%	-7.32%	-0.06%	-0.76%
S13	Low-medium	Landfill	Low	-0.55%	-8.45%	-0.40%	-2.66%	0.70%	1.55%	4.82%	0.92%	0.04%	-6.47%	1.34%	-1.15%
S14	Low-medium	Landfill	Medium	-0.55%	-8.45%	-0.40%	-2.66%	0.70%	1.55%	4.82%	0.92%	0.04%	-6.47%	1.34%	-1.15%
S15	Low-medium	Landfill	High	-0.55%	-8.45%	-0.40%	-2.66%	0.70%	1.55%	4.82%	0.92%	0.04%	-6.47%	1.34%	-1.15%
S16	Low-medium	Land application	Low	-0.48%	-8.39%	-1.54%	-2.39%	0.79%	1.62%	3.38%	1.26%	0.09%	-6.42%	0.32%	-0.91%
S17	Low-medium	Land application	Medium	-0.48%	-8.39%	-1.54%	-2.39%	0.79%	1.62%	3.38%	1.26%	0.10%	-6.42%	0.32%	-0.91%
S18	Low-medium	Land application	High	-0.48%	-8.39%	-1.54%	-2.39%	0.79%	1.62%	3.38%	1.26%	0.09%	-6.42%	0.32%	-0.91%
S19	Medium	Incineration	Low	-0.42%	-9.71%	-2.02%	-2.16%	0.89%	0.34%	2.88%	1.52%	0.14%	-7.61%	-0.15%	-0.72%
S20	Medium	Incineration	Medium	-0.42%	-9.71%	-2.02%	-2.16%	0.89%	0.34%	2.88%	1.52%	0.14%	-7.61%	-0.15%	-0.72%
S21	Medium	Incineration	High	-0.42%	-9.71%	-2.02%	-2.16%	0.89%	0.34%	2.88%	1.52%	0.14%	-7.61%	-0.15%	-0.72%
S22	Medium	Landfill	Low	-0.56%	-8.76%	-0.46%	-2.60%	0.72%	1.58%	4.90%	0.95%	0.02%	-6.76%	1.24%	-1.12%
S23	Medium	Landfill	Medium	-0.56%	-8.76%	-0.46%	-2.60%	0.72%	1.58%	4.90%	0.95%	0.02%	-6.76%	1.24%	-1.12%
S24	Medium	Landfill	High	-0.56%	-8.76%	-0.46%	-2.60%	0.72%	1.58%	4.90%	0.95%	0.02%	-6.76%	1.24%	-1.12%
S25	Medium	Land application	Low	-0.49%	-8.70%	-1.59%	-2.34%	0.80%	1.65%	3.43%	1.29%	0.08%	-6.71%	0.23%	-0.88%
S26	Medium	Land application	Medium	-0.49%	-8.70%	-1.59%	-2.34%	0.80%	1.65%	3.43%	1.29%	0.08%	-6.71%	0.23%	-0.88%
S27	Medium	Land application	High	-0.49%	-8.70%	-1.59%	-2.34%	0.80%	1.65%	3.43%	1.29%	0.08%	-6.71%	0.23%	-0.88%

GWP: Global warming potential, EP: Eutrophication potential, ET: Ecotoxicity, AP: Acidification potential

Table S16 Impact assessment results for Figure 3 – RP1-RP3 for wheat

				RP1				RP2				RP3			
**ID**	**Influent pollutant concentration**	**Sludge disposal**	**Carbon intensity of electricity**	GWP	EP	ET	AP	GWP	EP	ET	AP	GWP	EP	ET	AP
S01	Low	Incineration	Low	-6.14%	-21.89%	-16.23%	-3.25%	-0.36%	-31.39%	-13.58%	-4.50%	2.16%	-3.14%	3.51%	-0.26%
S02	Low	Incineration	Medium	-6.13%	-21.89%	-16.23%	-3.25%	-0.35%	-31.39%	-13.58%	-4.50%	2.15%	-3.14%	3.51%	-0.26%
S03	Low	Incineration	High	-6.15%	-21.89%	-16.23%	-3.25%	-0.37%	-31.39%	-13.58%	-4.50%	2.16%	-3.14%	3.51%	-0.26%
S04	Low	Landfill	Low	-6.59%	-21.41%	-14.65%	-3.57%	-0.67%	-30.64%	-10.95%	-4.90%	0.57%	0.81%	17.39%	-2.33%
S05	Low	Landfill	Medium	-6.58%	-21.41%	-14.65%	-3.57%	-0.65%	-30.64%	-10.95%	-4.89%	0.57%	0.81%	17.39%	-2.33%
S06	Low	Landfill	High	-6.59%	-21.41%	-14.65%	-3.57%	-0.67%	-30.64%	-10.95%	-4.90%	0.57%	0.81%	17.39%	-2.33%
S07	Low	Land application	Low	-6.49%	-21.38%	-15.91%	-3.41%	-0.52%	-30.59%	-12.86%	-4.66%	1.36%	1.05%	7.32%	-1.09%
S08	Low	Land application	Medium	-6.48%	-21.38%	-15.91%	-3.41%	-0.50%	-30.59%	-12.86%	-4.66%	1.36%	1.05%	7.32%	-1.09%
S09	Low	Land application	High	-6.49%	-21.38%	-15.91%	-3.41%	-0.52%	-30.59%	-12.86%	-4.66%	1.36%	1.05%	7.32%	-1.09%
S10	Low-medium	Incineration	Low	-5.36%	-42.94%	-12.38%	-2.61%	0.73%	-63.93%	-7.74%	-3.75%	2.33%	-3.28%	3.12%	-0.06%
S11	Low-medium	Incineration	Medium	-5.35%	-42.94%	-12.38%	-2.61%	0.74%	-63.93%	-7.74%	-3.75%	2.32%	-3.28%	3.12%	-0.06%
S12	Low-medium	Incineration	High	-5.36%	-42.94%	-12.38%	-2.61%	0.73%	-63.93%	-7.74%	-3.75%	2.33%	-3.28%	3.12%	-0.06%
S13	Low-medium	Landfill	Low	-5.47%	-42.68%	-11.46%	-2.75%	0.57%	-63.54%	-6.36%	-3.96%	0.65%	0.91%	17.82%	-2.25%
S14	Low-medium	Landfill	Medium	-5.46%	-42.68%	-11.46%	-2.75%	0.58%	-63.54%	-6.36%	-3.96%	0.64%	0.91%	17.82%	-2.25%
S15	Low-medium	Landfill	High	-5.47%	-42.68%	-11.46%	-2.75%	0.57%	-63.54%	-6.36%	-3.96%	0.65%	0.91%	17.82%	-2.25%
S16	Low-medium	Land application	Low	-5.41%	-42.66%	-12.13%	-2.66%	0.65%	-63.51%	-7.36%	-3.83%	1.48%	1.16%	7.15%	-0.94%
S17	Low-medium	Land application	Medium	-5.41%	-42.66%	-12.13%	-2.66%	0.66%	-63.51%	-7.36%	-3.83%	1.48%	1.16%	7.15%	-0.94%
S18	Low-medium	Land application	High	-5.42%	-42.66%	-12.13%	-2.66%	0.65%	-63.51%	-7.36%	-3.83%	1.48%	1.16%	7.15%	-0.94%
S19	Medium	Incineration	Low	-4.25%	-65.11%	-7.38%	-1.74%	2.04%	-90.89%	-1.67%	-2.82%	2.37%	-3.31%	3.01%	-0.01%
S20	Medium	Incineration	Medium	-4.24%	-65.11%	-7.38%	-1.74%	2.04%	-90.89%	-1.67%	-2.82%	2.37%	-3.31%	3.01%	-0.01%
S21	Medium	Incineration	High	-4.25%	-65.11%	-7.38%	-1.74%	2.04%	-90.89%	-1.67%	-2.82%	2.38%	-3.31%	3.01%	-0.01%
S22	Medium	Landfill	Low	-4.25%	-65.09%	-7.32%	-1.74%	2.01%	-90.81%	-1.37%	-2.87%	0.67%	0.93%	17.94%	-2.23%
S23	Medium	Landfill	Medium	-4.25%	-65.09%	-7.32%	-1.75%	2.01%	-90.81%	-1.37%	-2.87%	0.66%	0.93%	17.94%	-2.23%
S24	Medium	Landfill	High	-4.26%	-65.09%	-7.32%	-1.75%	2.01%	-90.81%	-1.37%	-2.87%	0.67%	0.93%	17.94%	-2.23%
S25	Medium	Land application	Low	-4.25%	-65.09%	-7.37%	-1.74%	2.03%	-90.80%	-1.59%	-2.84%	1.52%	1.19%	7.11%	-0.90%
S26	Medium	Land application	Medium	-4.25%	-65.09%	-7.37%	-1.74%	2.03%	-90.80%	-1.59%	-2.84%	1.51%	1.19%	7.11%	-0.90%
S27	Medium	Land application	High	-4.25%	-65.09%	-7.37%	-1.74%	2.02%	-90.80%	-1.59%	-2.84%	1.52%	1.19%	7.11%	-0.90%

GWP: Global warming potential, EP: Eutrophication potential, ET: Ecotoxicity, AP: Acidification potential

Table S17 Impact assessment results for Figure 3 – RP4-RP6 for wheat

				RP4				RP5				RP6			
**ID**	**Influent pollutant concentration**	**Sludge disposal**	**Carbon intensity of electricity**	GWP	EP	ET	AP	GWP	EP	ET	AP	GWP	EP	ET	AP
S01	Low	Incineration	Low	-1.29%	-17.55%	-5.07%	-6.81%	7.31%	0.92%	17.62%	4.73%	4.58%	-11.57%	11.49%	-0.79%
S02	Low	Incineration	Medium	-1.27%	-17.55%	-5.07%	-6.81%	7.31%	0.92%	17.62%	4.73%	4.60%	-11.57%	11.49%	-0.79%
S03	Low	Incineration	High	-1.30%	-17.55%	-5.07%	-6.81%	7.31%	0.92%	17.62%	4.73%	4.57%	-11.57%	11.49%	-0.79%
S04	Low	Landfill	Low	-2.63%	-14.21%	6.68%	-8.56%	5.88%	4.48%	30.12%	2.87%	3.38%	-8.59%	21.96%	-2.35%
S05	Low	Landfill	Medium	-2.62%	-14.21%	6.68%	-8.56%	5.88%	4.48%	30.12%	2.87%	3.40%	-8.59%	21.96%	-2.35%
S06	Low	Landfill	High	-2.64%	-14.21%	6.68%	-8.56%	5.88%	4.48%	30.12%	2.87%	3.37%	-8.59%	21.96%	-2.35%
S07	Low	Land application	Low	-1.97%	-14.01%	-1.85%	-7.51%	6.60%	4.69%	21.05%	3.99%	3.98%	-8.41%	14.36%	-1.42%
S08	Low	Land application	Medium	-1.95%	-14.01%	-1.85%	-7.51%	6.59%	4.69%	21.05%	3.99%	4.00%	-8.41%	14.36%	-1.41%
S09	Low	Land application	High	-1.97%	-14.01%	-1.85%	-7.51%	6.60%	4.69%	21.05%	3.99%	3.97%	-8.41%	14.36%	-1.42%
S10	Low-medium	Incineration	Low	-3.66%	-28.73%	-12.93%	-7.68%	7.71%	1.01%	18.59%	5.14%	1.36%	-22.36%	-0.42%	-2.62%
S11	Low-medium	Incineration	Medium	-3.65%	-28.73%	-12.93%	-7.68%	7.70%	1.01%	18.59%	5.14%	1.38%	-22.36%	-0.42%	-2.62%
S12	Low-medium	Incineration	High	-3.66%	-28.73%	-12.93%	-7.68%	7.71%	1.01%	18.59%	5.14%	1.36%	-22.36%	-0.42%	-2.62%
S13	Low-medium	Landfill	Low	-4.84%	-25.79%	-2.60%	-9.22%	6.21%	4.74%	31.69%	3.19%	0.31%	-19.75%	8.78%	-3.99%
S14	Low-medium	Landfill	Medium	-4.83%	-25.79%	-2.60%	-9.22%	6.21%	4.74%	31.69%	3.19%	0.32%	-19.75%	8.78%	-3.99%
S15	Low-medium	Landfill	High	-4.84%	-25.79%	-2.60%	-9.22%	6.21%	4.74%	31.69%	3.19%	0.31%	-19.75%	8.78%	-3.99%
S16	Low-medium	Land application	Low	-4.25%	-25.61%	-10.10%	-8.30%	6.96%	4.96%	22.18%	4.36%	0.84%	-19.59%	2.11%	-3.17%
S17	Low-medium	Land application	Medium	-4.24%	-25.61%	-10.10%	-8.30%	6.95%	4.96%	22.18%	4.36%	0.85%	-19.59%	2.11%	-3.17%
S18	Low-medium	Land application	High	-4.25%	-25.61%	-10.10%	-8.30%	6.96%	4.96%	22.18%	4.36%	0.84%	-19.59%	2.11%	-3.17%
S19	Medium	Incineration	Low	-3.74%	-29.66%	-13.27%	-7.49%	7.84%	1.04%	18.92%	5.28%	1.23%	-23.23%	-1.00%	-2.51%
S20	Medium	Incineration	Medium	-3.73%	-29.66%	-13.27%	-7.49%	7.84%	1.04%	18.92%	5.28%	1.24%	-23.23%	-1.00%	-2.51%
S21	Medium	Incineration	High	-3.74%	-29.66%	-13.27%	-7.50%	7.84%	1.04%	18.92%	5.28%	1.23%	-23.23%	-1.00%	-2.51%
S22	Medium	Landfill	Low	-4.91%	-26.74%	-3.02%	-9.02%	6.32%	4.82%	32.23%	3.30%	0.19%	-20.64%	8.13%	-3.88%
S23	Medium	Landfill	Medium	-4.90%	-26.74%	-3.02%	-9.02%	6.32%	4.82%	32.23%	3.30%	0.20%	-20.64%	8.13%	-3.88%
S24	Medium	Landfill	High	-4.92%	-26.74%	-3.02%	-9.02%	6.32%	4.82%	32.23%	3.30%	0.19%	-20.64%	8.13%	-3.88%
S25	Medium	Land application	Low	-4.33%	-26.56%	-10.46%	-8.11%	7.08%	5.05%	22.57%	4.49%	0.71%	-20.48%	1.51%	-3.06%
S26	Medium	Land application	Medium	-4.32%	-26.56%	-10.46%	-8.10%	7.08%	5.05%	22.57%	4.49%	0.72%	-20.48%	1.51%	-3.06%
S27	Medium	Land application	High	-4.33%	-26.56%	-10.46%	-8.11%	7.08%	5.05%	22.57%	4.49%	0.71%	-20.48%	1.51%	-3.06%

GWP: Global warming potential, EP: Eutrophication potential, ET: Ecotoxicity, AP: Acidification potential

S4. Monte Carlo simulation

Monte Carlo simulation was performed on the maize production system by propagating parameter uncertainty (Table S17) for all recovery pathways and their scenarios. The uncertainties within crop production system are excluded because the comparison is between the baseline pathway and the recovery pathways, of which they share the same crop production system. Because of the simulation size, infrastructure processes have also been excluded. Each simulation consists of 1000 runs.

Table S18 Parameters used for Monte Carlo Simulation

Parameters	Value	Distribution	SD^2	Lower	Upper	Remark
**Universal category**						
Water content of dewatered digested sludge	0.72	Lognormal	1.25	-	-	-
RP1 P bioavailability	1	Triangle	-	0.8	1	-20%
RP2 P bioavailability	0.7	Triangle	-	0.56	0.7	-20%
RP3 P bioavailability	0.9	Triangle	-	0.72	0.9	-20%
RP4 P bioavailability	0.9	Triangle	-	0.72	0.9	-20%
RP5 P bioavailability	1	Triangle	-	0.8	1	-20%
RP6 P bioavailability	1	Triangle	-	0.8	1	-20%
Transport distance for phosphorus recovered product (km)	400	Triangle	-	200	600	±50%
RP1 P content (% dry matter)	0.126	Triangle	-	0.1008	0.126	-20%
RP2 P content (% dry matter)	0.2	Triangle	-	0.16	0.2	-20%
RP3 P content (% dry matter)	0.074	Triangle	-	0.0592	0.074	-20%
RP4 P content (% dry matter)	0.074	Triangle	-	0.0592	0.074	-20%
RP5 P content (% dry matter)	0.087	Triangle	-	0.0696	0.087	-20%
RP6 P content (% dry matter)	0.087	Triangle	-	0.0696	0.087	-20%
**Scenario category**						
Biogas production	Vary	Lognormal	1.25	-	-	
Magnesium hydroxide usage	Vary	Lognormal	1.5	-	-	MgO process for $Mg(OH)_2$
Sodium hydroxide usage	Vary	Lognormal	1.25	-	-	
Average aeration energy consumption	Vary	Lognormal	1.25	-	-	
Average pumping energy consumption	Vary	Lognormal	1.25	-	-	
Emissions to water: Phosphorus, total	Vary	Lognormal	1.25	-	-	
Emissions to water: Nitrogen, total	Vary	Lognormal	1.25	-	-	
Emissions to water: COD, Chemical Oxygen Demand	Vary	Lognormal	1.25	-	-	
Sludge for disposal	Vary	Lognormal	1.25	-	-	
All remaining foreground inventory	Vary	Lognormal	1.25	-	-	
**Background inventory category**						
Phosphate fertiliser, as P2O5 {GLO}| market for | Conseq, U						Using unit process (Ecoinvent) with default upstream uncertainty parameters
Ammonium nitrate, as N {GLO}| market for | Conseq, U						
Nitrogen fertiliser, as N {GLO}| market for | Conseq, U						
Methane, 96% by volume, from biogas, low pressure, at user {RoW}| production | Conseq, U						
Magnesium oxide {GLO}| market for | Conseq, U						
Sodium hydroxide, without water, in 50% solution state {GLO}| market for | Conseq, U						
Lime, hydrated, loose weight {RoW}| market for lime, hydrated, loose weight | Conseq, U						
Transport, freight, lorry, unspecified {GLO}| market group for transport, freight, lorry, unspecified | Conseq, U						
Electricity, medium voltage {NPCC, US only}| market for | Conseq, U						
Electricity, medium voltage {WECC, US only}| market for | Conseq, U						
Electricity, medium voltage {MRO, US only}| market for | Conseq, U						
Digester sludge {GLO}| treatment of digester sludge, municipal incineration | Conseq, U						
Nitrogen fertiliser, as N {RoW}| monoammonium phosphate production | APOS, U						
Nitrogen fertiliser, as N {RoW}| diammonium phosphate production | APOS, U						
Phosphate fertiliser, as P2O5 {RER}| single superphosphate production | Conseq, U						
Carbon dioxide, in air						
All remaining background inventory, except crop production processes and infrastructure processes						

The Monte Carlo simulation results are presented as the probability of each resource recovery pathway having lower impact potential than the baseline pathway (Figure S2). For instance, in the case of global warming potential, RP1 has nearly 100% certainty that the recovery pathway (struvite) across all studied scenarios has lower global warming potential than the baseline pathway. In most cases, the certainty depends a lot on the scenarios (influent pollutant concentration, sludge disposal method, and carbon intensity of grid electricity), i.e., the points are spreading out in clusters.

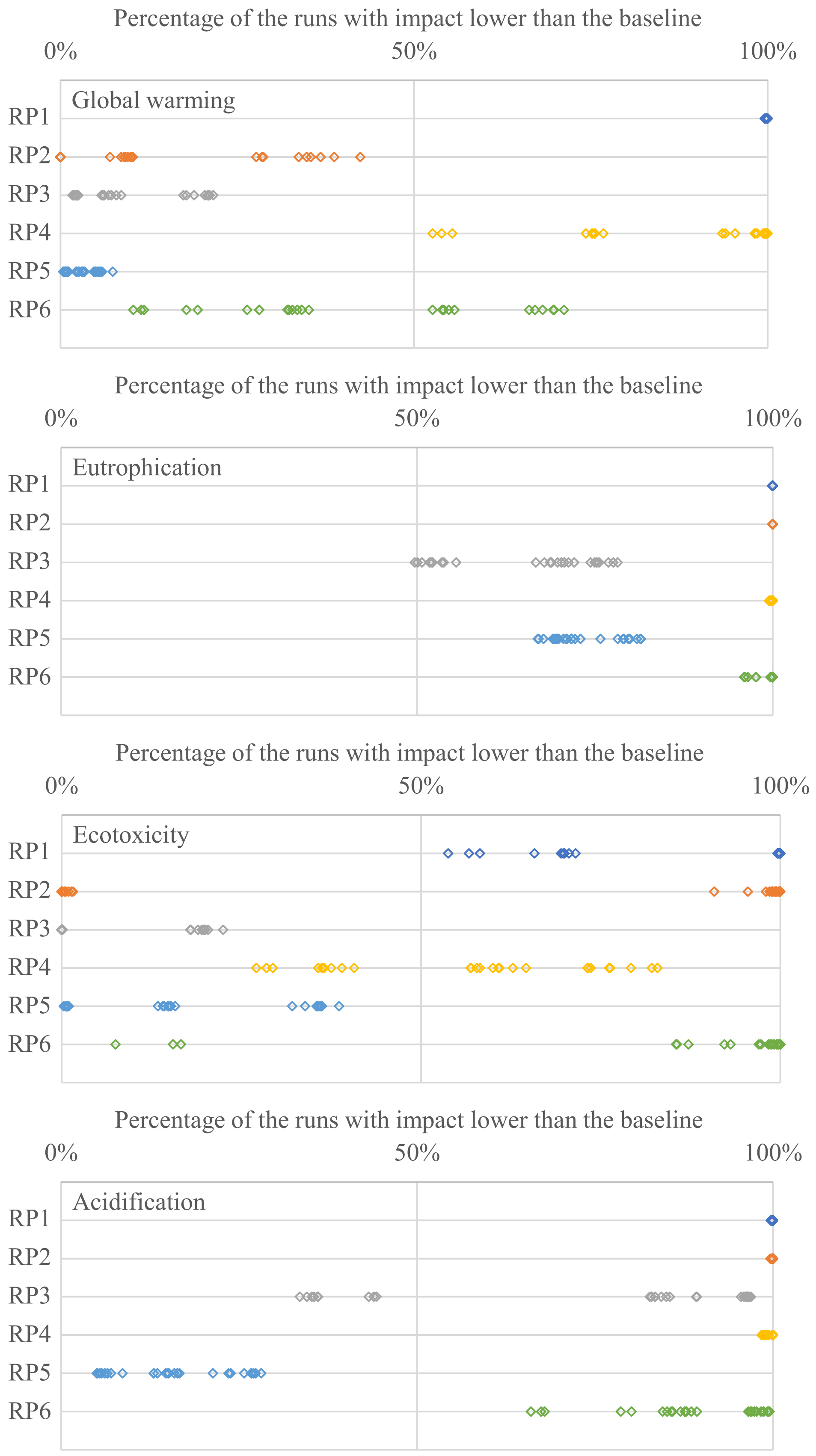

Figure S3 Uncertainty analysis with Monte Carlo simulation. Each point shows the percentage of the runs (within a 1000-run Monte Carlo simulation) in a scenario with the impact of a given RP lower than that of the baseline.

S5. Sensitivity analysis

In this sensitivity analysis, we assessed how the changes in each of the following inputs would influence the "default" results for maize production relative to the baseline. The default scenario is low-medium influent pollutant concentration, sludge landfill, medium carbon intensity of electricity, 400 km product transportation distance, and RP1 bioavailability of 1, RP2 bioavailability of 0.7, RP3-RP4 bioavailability of 0.9 and RP5-RP6 bioavailability of 1. Bioavailability of recovered products has strong influence across all RPs, while sludge disposal method influences more on the ash-based recovery pathways (RP3-RP6).

Table S19 Sensitivity analysis relative to the "default" results for maize production

		Low influent pollutant concentration	Medium influent pollutant concentration	Sludge incineration	Land application of sludge	Low carbon intensity of electricity	High carbon intensity of electricity	Bioavailability (-50%)	Product transportation distance (+100%)
RP1	Global warming	32.9%	-35.4%	-3.1%	-1.5%	0.3%	0.3%	3.7%	-6.2%
	Eutrophication	-50.1%	52.8%	0.6%	0.0%	0.0%	0.0%	97.6%	0.0%
	Ecotoxicity	39.1%	-50.8%	11.3%	8.2%	0.0%	0.0%	37.9%	-0.5%
	Acidification	24.1%	-29.3%	-4.0%	-2.4%	0.0%	0.0%	15.1%	-2.6%
RP2	Global warming	-212.7%	246.2%	27.2%	13.6%	-1.4%	-1.8%	301.5%	30.5%
	Eutrophication	-51.8%	42.9%	0.6%	0.0%	0.0%	0.0%	98.6%	0.0%
	Ecotoxicity	72.2%	-78.4%	21.7%	15.8%	0.0%	0.0%	62.7%	-0.5%
	Acidification	23.7%	-27.5%	-5.2%	-3.1%	0.0%	0.0%	1.9%	-2.2%
RP3	Global warming	-12.0%	3.4%	261.3%	130.3%	0.6%	0.8%	276.1%	64.3%
	Eutrophication	-10.8%	3.1%	-461.6%	27.8%	0.0%	0.0%	196.9%	2.8%
	Ecotoxicity	-2.4%	0.7%	-82.5%	-59.9%	0.0%	0.0%	113.1%	0.4%
	Acidification	3.4%	-1.0%	-97.2%	-58.3%	0.0%	0.0%	-72.1%	-8.8%
RP4	Global warming	-45.8%	1.6%	-24.4%	-12.2%	0.2%	0.2%	76.4%	-8.2%
	Eutrophication	-44.9%	3.7%	11.4%	-0.7%	0.0%	0.0%	96.6%	-0.1%
	Ecotoxicity	-356.6%	15.9%	396.6%	287.8%	0.0%	0.0%	10.1%	-2.7%
	Acidification	-7.2%	-2.1%	-16.7%	-10.0%	0.0%	0.0%	58.1%	-2.6%
RP5	Global warming	-5.2%	1.8%	24.1%	12.0%	0.0%	0.0%	118.5%	4.7%
	Eutrophication	-5.5%	1.9%	-78.7%	4.7%	0.0%	0.0%	118.6%	0.3%
	Ecotoxicity	-5.0%	1.7%	-41.3%	-30.0%	0.0%	0.0%	107.6%	0.1%
	Acidification	-10.0%	3.4%	61.3%	36.8%	0.0%	0.0%	221.3%	6.0%
RP6	Global warming	947.9%	-39.3%	324.0%	161.5%	-3.3%	-4.1%	448.3%	94.2%
	Eutrophication	-56.5%	4.5%	13.3%	-0.8%	0.0%	0.0%	95.5%	-0.1%
	Ecotoxicity	150.1%	-7.4%	-104.8%	-76.0%	0.0%	0.0%	126.6%	0.8%
	Acidification	-41.1%	-2.9%	-34.4%	-20.6%	0.0%	0.0%	2.4%	-4.5%

References

1. Solon, K.; Flores-Alsina, X.; Kazadi Mbamba, C.; Ikumi, D.; Volcke, E. I. P.; Vaneeckhaute, C.; Ekama, G.; Vanrolleghem, P. A.; Batstone, D. J.; Gernaey, K. V.; Jeppsson, U., Plant-wide modelling of phosphorus transformations in wastewater treatment systems: Impacts of control and operational strategies. *Water Res.* **2017,** *113*, 97-110.
2. Volcke, E. I. P., Solon, K., Comeau, Y., Henze, M, Wastewater characteristics. In *2nd Ed Biological Wastewater Treatment: Principles, Modelling and Design*, Chen, G. H.; van Loosdrecht, M. C. M.; Ekama, G. A.; Brdjanovic, D., Eds. IWA Publishing: London, UK, 2020.
3. Tonini, D.; Saveyn, H. G. M.; Huygens, D., Environmental and health co-benefits for advanced phosphorus recovery. *Nature Sustain.* **2019,** *2*, (11), 1051-1061.
4. Terman, G. L.; Bouldin, D. R.; Lehr, J. R., Calcium Phosphate Fertilizers: I. Availability to Plants and Solubility in Soils Varying in pH. *Soil Science Society of America Journal* **1958,** *22*, (1), 25-29.
5. Römer, W.; Steingrobe, B., Fertilizer effect of phosphorus recycling products. *Sustainability* **2018,** *10*, (4), 1166.
6. Alanya, S.; Dewulf, J.; Duran, M., Comparison of Overall Resource Consumption of Biosolids Management System Processes Using Exergetic Life Cycle Assessment. *Environ. Sci. Technol.* **2015,** *49*, (16), 9996-10006.
7. Wernet, G.; Bauer, C.; Steubing, B.; Reinhard, J.; Moreno-Ruiz, E.; Weidema, B., The ecoinvent database version 3 (part I): overview and methodology. *The International Journal of Life Cycle Assessment* **2016,** *21*, (9), 1218-1230.
